# Development of a transdiagnostic, low-intensity, psychological intervention for common adolescent mental health problems in Indian secondary schools

**DOI:** 10.1016/j.brat.2019.103439

**Published:** 2020-07

**Authors:** Daniel Michelson, Kanika Malik, Madhuri Krishna, Rhea Sharma, Sonal Mathur, Bhargav Bhat, Rachana Parikh, Kallol Roy, Akankasha Joshi, Rooplata Sahu, Bhagwant Chilhate, Maya Boustani, Pim Cuijpers, Bruce Chorpita, Christopher G. Fairburn, Vikram Patel

**Affiliations:** a School of Psychology, University of Sussex, Brighton, UK; b Sangath, Goa and New Delhi, India; c Department of Clinical Psychology, Vrije Universiteit, Amsterdam, Netherlands; d Shree Krishna Hospital, Gujarat, India; e Department of Psychology, Loma Linda University, Los Angeles, USA; f Department of Psychology, University of California at Los Angeles, Los Angeles, USA; g Department of Psychiatry, University of Oxford, Oxford, UK; h Department of Global Health and Social Medicine, Harvard Medical School, Boston, USA; i Harvard TH Chan School of Public Health, Boston, USA

**Keywords:** Adolescents, Mental health, Transdiagnostic, Schools, Psychological intervention, India, PRIDE, PremIum for aDolEscents

## Abstract

**Background:**

The PRIDE programme aims to establish a suite of transdiagnostic psychological interventions organised around a stepped care system in Indian secondary schools. This paper describes the development of a low-intensity, first-line component of the PRIDE model.

**Method:**

Contextual and global evidence informed an intervention ‘blueprint’ with problem solving as the primary practice element. Successive iterations were tested and modified across two pilot cohort studies (N = 45; N = 39). Participants were aged 13–20 years and presenting with elevated mental health symptoms in New Delhi schools.

**Results:**

The first iteration of the intervention, based on a guided self-help modality, showed promising outcomes and user satisfaction when delivered by psychologists. However, delivery was not feasible within the intended 6-week schedule, and participants struggled to use materials outside ‘guidance’ sessions. In Pilot 2, a modified counsellor-led problem-solving intervention was implemented by less experienced counsellors over a 3–4 week schedule. Outcomes were maintained, with indications of enhanced feasibility and acceptability. High demand was observed across both pilots, leading to more stringent eligibility criteria and a modified sensitisation plan.

**Discussion:**

Findings have shaped a first-line intervention for common adolescent mental health problems in low-resource settings. A forthcoming randomised controlled trial will test its effectiveness.

## Highlights

• PRIDE is developing a transdiagnostic, stepped care programme in Indian schools.
• Formative evidence supported problem solving as a first-line ‘Step 1’ intervention.
• The format was modified from guided self-help to an active therapy after piloting.
• The optimised intervention showed promising impact, acceptability and feasibility.

## 1 Introduction

### 1.1 Background

Early intervention for youth mental health problems is a global priority (Holmes et al., 2018). Mental health conditions typically have their onset in the first two decades of life and are leading causes of social disability in the adolescent demographic worldwide (Davidson, Grigorenko, Boivin, Rapa, & Stein, 2015). Around 10% of adolescents aged 10–19 years have a clinically diagnosable mental disorder, with anxiety, depression and conduct difficulties together accounting for over 75% of the total mental health burden in this age group (Erskine et al., 2015). If untreated, these common mental health presentations can exert serious detrimental effects on young people's developmental progress, family life and educational achievement, with long-term implications for poor health, social exclusion and lower economic activity in adulthood (St John, Leon, & McCulloch, 2005). There are also strong links between youth mental health problems and suicide, which represents a leading cause of premature mortality throughout the world (World Health Organization, 2014a).

The burden of adolescent mental disorders falls mostly on low- and middle-income countries (LMICs). About 250 million adolescents – one-fifth of the world's total adolescent population – reside in India alone. Many adolescents in India and other LMICs are exposed to multiple psychosocial adversities, especially in deprived urban areas (Fisher et al., 2011; Viner et al., 2012), posing cumulative risks for onset and persistence of mental disorders (Green et al., 2010; McLaughlin et al., 2010). Wider social inequalities increase vulnerability even further by limiting participation in protective educational, family and peer activities (Viner et al., 2012). Correspondingly, studies conducted in urban India have indicated that one in five adolescents endure high levels of stress in their daily lives (Kumar & Akoijam, 2017; Mathew, Khakha, Qureshi, Sagar, & Khakha, 2015), whereas the most recent National Mental Health Survey in India has reported prevalence estimates of 13.5% for adolescent mental disorders in urban metropolitan areas and 6.9% in rural areas (Gururaj et al., 2016). At the same time, access to mental health care is extremely restricted. Only 1.93 mental health workers are found in India per 100,000 population (World Health Organization, 2018a), compared with 71.7 per 100,000 in high-income countries (World Health Organization, 2018b); a tiny fraction of these workers are specifically oriented towards adolescent mental health needs. Such dimensions underscore the major challenges and opportunities that exist for improving youth mental health and related outcomes at scale.

Context-specific research is urgently needed to guide the efforts of service planners, developers and providers in India and other LMICs. Existing studies on adolescent mental health interventions in LMICs have largely focused on either generic mental health promotion for younger children, often in schools, or psychological treatments for highly selected trauma-affected populations (Barry, Clarke, Jenkins, & Patel, 2013; Klasen & Crombag, 2013). Far less attention has been devoted to psychotherapies for mixed emotional and behavioural difficulties in general adolescent populations, even though this pattern represents the majority of ‘real-world’ case mix (Weisz, Krumholz, Santucci, Thomassin, & Ng, 2015).

In high-income country contexts, transdiagnostic interventions have attracted interest following from evidence that some psychological processes implicated in the maintenance of psychopathology are shared across certain mental disorders (Ehrenreich-May & Chu, 2013; Levy, Hawes, & Johns, 2015). Moreover, many of the constituent ‘practice elements’ of evidence-based intervention protocols appear to be relevant to a wide variety of child and adolescent problems and disorders (i.e., certain elements are not restricted to effective treatments for a specific type of problem or disorder; Chorpita & Daleiden, 2009). Transdiagnostic principles are also aligned with shifting conceptualisations of mental health towards more dimensional models of symptoms and impairment (LeBeau, Bögels, Möller, & Craske, 2015; McGorry, Nelson, Goldstone, & Yung, 2010). Recent studies, primarily from adult populations and focusing on anxiety and depression, suggest that transdiagnostic treatments may be at least as effective as disorder-specific approaches (Newby, McKinnon, Kuyken, Gilbody, & Dalgleish, 2015), and possibly more suitable for scaling up (Creed et al., 2016). Secondary prevention programs have also been piloted for nonspecific and subthreshold mental health presentations, with promising results (Brown et al., 2018; Topper, Emmelkamp, Watkins, & Ehring, 2017). A further strand of research, centred on humanitarian contexts, has pointed to the feasibility of transdiagnostic interventions in LMICs (Murray et al., 2018; Murray & Jordans, 2016). However, key questions relate to the generalisability of these findings to routine global settings where demand and supply for mental health care are strongly influenced by local culture and resource characteristics (Belkin et al., 2011; Lewis-Fernández & Aggarwal, 2013; Padmanathan & De Silva, 2013).

### 1.2 The PRIDE programme

PRIDE (PremIum for aDolEscents) aims to develop and test a suite of evidence-based interventions addressing the major share of the adolescent mental health burden (i.e., anxiety, depression and conduct difficulties) in India. This builds on a robust methodology that was developed as part of the Programme for Effective Mental Health Interventions in Under-resourced settings (PREMIUM) from 2010 to 2015 (Nadkarni et al., 2017; Patel et al., 2017). This established a systematic phased approach for psychological treatment development and evaluation in under-resourced settings, involving: (i) formative research to inform initial intervention modelling; (ii) field testing and refinement in pilot evaluations; (iii) a definitive randomised controlled trial (Vellakkal & Patel, 2015).

The current paper encompasses phases (i) and (ii) with the aim of developing a first-line, low-intensity transdiagnostic intervention (‘Step 1’) for school-going adolescents with elevated mental health symptoms. The design of a high-intensity modular treatment (‘Step 2’), which forms a second component of a sequential stepped care architecture, will be described in detail elsewhere. The phased research programme will culminate in a series of randomised controlled trials addressing each step individually and in combination (i.e. completing phase iii as above) (Parikh et al., 2019(Parikh et al., 2019).

Here we describe the lessons from formative and pilot studies within a single manuscript in order to provide a comprehensive narrative about the iterative process of developing the Step 1 intervention. This took place over a period of 2 and a half years and, following the methodology derived from PREMIUM, was shaped by multiple sources of context-specific evidence on population needs and resources, key international empirical and theoretical literature, and extended piloting. The depth and breadth of the design process offered the potential to arrive at an optimised and scalable intervention with the goal of achieving theorised individual outcomes and large-scale impact.

## 2 Overview

### 2.1 Research design

**Fig. 1:**
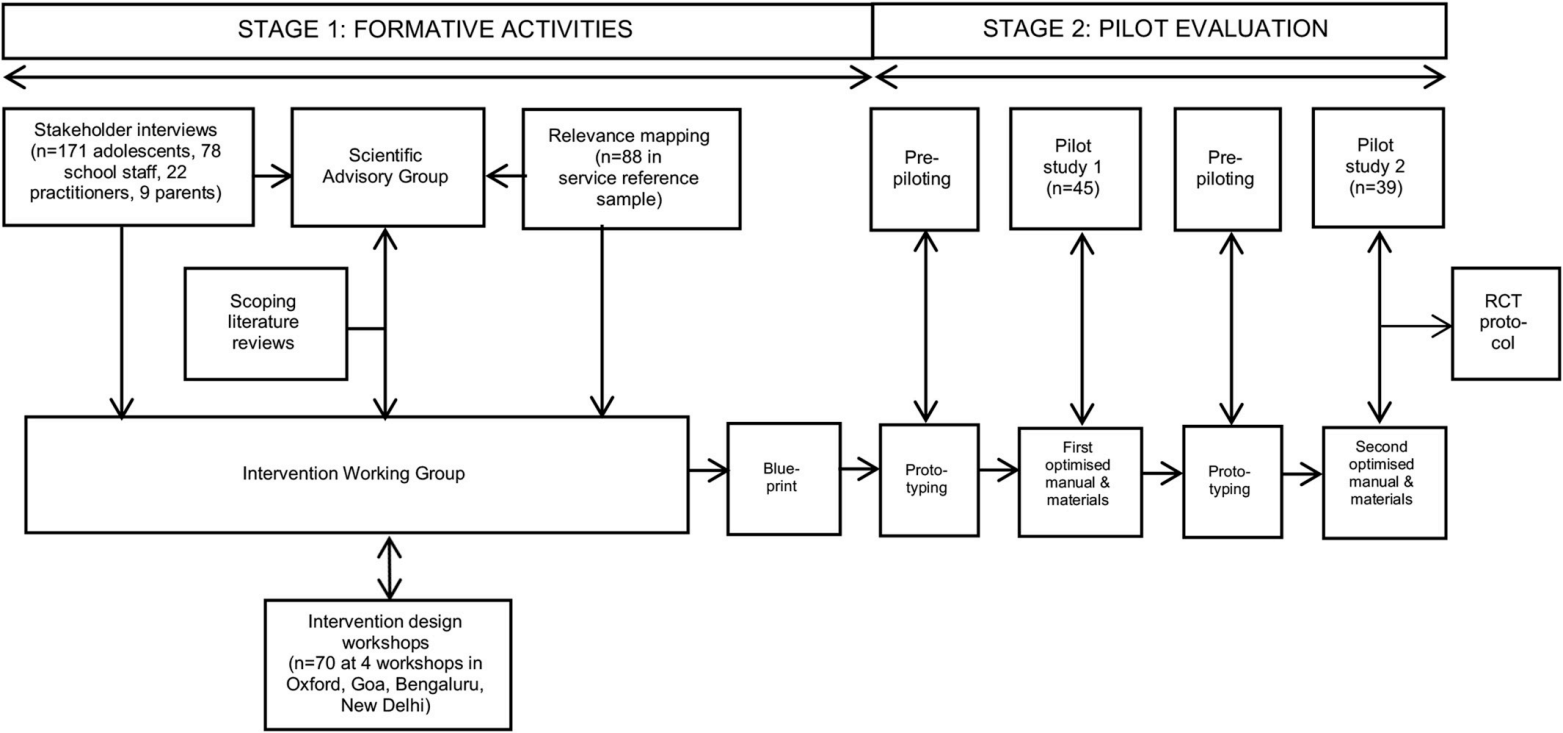
Schematic of intervention development process and outputs.

An iterative phased approach was used to model and then test successive prototypes of the intervention in two linked pilot studies using a prospective cohort design (see Fig. 1). The objectives of the formative and pilot studies were to:

(i) develop the provisional architecture, theoretical framework and practice materials for a school-based intervention that is intended to reduce symptom severity and improve associated functional impairment among adolescents with common mental health problems in India
(ii) evaluate the acceptability of intervention delivery (i.e., the extent to which the intended participants were actively engaged in and receptive to each iteration, and the factors that impeded or supported their optimal use of the intervention);
(iii) evaluate the feasibility of intervention delivery (i.e., the extent to which each iteration was delivered as planned, and the factors that impeded or supported optimal delivery);
(iv) evaluate potential for impact (i.e., the extent to which each iteration affected theorised and unintended outcomes); and
(v) refine intervention parameters based on emergent findings.

### 2.2 Setting

Formative activities were initiated in January 2016 and completed primarily in Goa (the country's most highly urbanised state) and New Delhi (India's capital). Additional intervention design workshops were completed in early 2016 with experts in Oxford, UK and Bengaluru, India. The two pilot studies were conducted in Government-run, Hindi-medium secondary schools in New Delhi during successive academic years, starting in the summer of 2017. Individual schools were purposively selected in consultation with the local Department of Education, focusing on low-income communities and schools without existing counselling provision. Pilot 1 took place in three same-sex schools (1 all-girls and 2 all-boys schools); three additional schools (1 all-girls, 1 all-boys and 1 co-educational school) were involved in Pilot 2. School populations (spanning grades 6–12) ranged in size from 2700 to 3073.

We obtained approvals from the Indian Council of Medical Research and the Institutional Review Boards of the sponsor (Harvard Medical School); a collaborating academic partner (London School of Hygiene and Tropical Medicine); and the two implementing organisations in India (Public Health Foundation of India and Sangath). Informed consent was gathered from all adolescents aged 18 years or older, with informed assent and corresponding parental consent obtained for younger adolescents.

### 2.3 Data collection

Formative and pilot data sources are summarised in Table 1, Table 2 respectively. Given the paucity of validated instruments in India and other LMICs, standardised measures were selected based on their reliability, validity and clinical utility in other adolescent mental health contexts. These were subjected to forward/backward translations, unless a Hindi version was already available. Case records (paper and digital) and referral logs were used to extract quantitative process indicators on intervention delivery. Modifications were made to the case records after Pilot 1 to streamline data collection and interpretation; for example, closed categories, arrived through content analysis of free-text responses in Pilot 1, replaced free-text fields to assess engagement with intervention materials and procedures in Pilot 2.

**Table 1:** Formative data sources and findings.

Source/Purpose	Key findings
**Intervention design workshops:** To align intervention design parameters with key theoretical principles, recent empirical evidence and national/international best practice; and refine formative research questions and methods	• Wide age range and multiple referral routes can maximise coverage, impact and buy-in from schools • Delivery in schools can reduce external structural barriers to accessing psychological interventions, but other constraints may be faced due to daily timetable (e.g., 40-min class periods), vacations and exam periods • Challenges of implementing systematic mental health screening in schools require brief, ecologically valid assessment tools, focused on symptom-based/functional dimensions rather than discrete diagnostic categories • Transdiagnostic ‘elements-based’ intervention design may have particular utility in designing parsimonious treatment packages in low-resource contexts • Public health impact may be strengthened through a stepped care approach that delivers a low-intensity intervention across diverse presentations, followed by a high-intensity treatment for non-responders that is tailored to specific problem profiles (e.g., by selecting/sequencing discrete treatment modules) • A relatively brief psychological intervention, focused on ‘here and now’ strategies, may be favoured by adolescents and is consistent with the requirements of a low-intensity first-line intervention • Simplified decision rules are needed to facilitate delivery by non-specialists • Digital delivery platforms and parental involvement should be explored further
**Scoping literature reviews:** To align intervention design parameters and decisions with the global evidence base	• Emerging support for transdiagnostic mechanisms in onset, maintenance and treatment of common mental health problems • Substantial support for stress-coping principles and their applications in cognitive and behavioural therapies, including self-help approaches • Self-help is most effective when provided with guidance, which may be delivered in various formats • Adolescents' may prefer practical coping strategies that fit with developmental drive for self-determination • Stepped care models, linked to measurement feedback systems, can maximise effectiveness and efficiency of treatments by optimising resource allocation • Task-sharing approaches with non-specialists have been effective in a growing number of psychological treatment trials, particularly in low-resource contexts, when accompanied by adequate supervision • Peer-led supervision approaches have potential utility as part of task sharing
**Local stakeholder interviews:** To obtain contextually sensitive evidence about types and causes of common adolescent mental health problems; adaptive and maladaptive coping strategies; knowledge and attitudes towards help-seeking; and preferences and priorities for psychological support	• Adolescent help seeking is often driven by psychosocial stressors rather than overt psychiatric symptoms • Adolescents prioritise concrete, practical tips for problem resolution • Self-help is largely unfamiliar as a concept among the target population; face-to-face guidance may help to explain materials and strengthen engagement • Digital technology is appealing for adolescents (especially use of films/animations), but there is limited access to personal devices and distribution of handsets could arouse suspicion from parents, teachers and peers • Adolescents are generally opposed to parental/teacher involvement in counselling, whereas significant adults wish to be kept informed about problems and progress • School counsellors are less likely to have strong stigmatising connotations, relative to psychiatrists and other clinic-based mental health service providers • Mental health literacy of staff and support for service implementation can vary greatly between schools
**Relevance mapping:** To identify evidence-based practice elements from global research literature that can be applied most widely to presenting problems in the target population	• Relative to other individual practice elements, problem solving offers the most parsimonious coverage to the range of presenting problems likely to occur in the target population • This includes a substantial proportion of psychosocial problems that do not correspond precisely to standardised mental health symptom inventories, but may nevertheless be associated with elevated distress and functional impairment • The full international literature, as well as the subset of studies in non-Western contexts, all showed similar support for problem solving

**Table 2:** Clinical and evaluative measures used in pilot studies.

Type	Description	Administration in Pilot 1	Administration in Pilot 2
Outcome measures and clinical tools	The Strengths and Difficulties Questionnaire (SDQ; Goodman, Ford, Simmons, Gatward, & Meltzer, 2000) is a 25-item self-report measure of youth mental health. A Total Difficulties score is derived and an Impact Supplement measures associated distress and functional impairment, with an additional descriptive item on chronicity of difficulties. Borderline and abnormal cut-offs were calculated based on the top 20% and 10% of scores obtained for a normative reference sample in India (Bhola, Sathyanarayanan, Rekha, Daniel, & Thomas, 2016)	Counsellor at baseline; researcher at end of intervention	Researcher at baseline/end of intervention
	The SDQ Session by Session (SxS) (Hall et al., 2014, 2015) measure is a modified form of the SDQ Impact Supplement that is intended for intervention progress monitoring. Self-rated items assess adolescents' perceptions of recent improvement, impacts of problems on everyday life in the present and anticipated improvement in the future.	Counsellor at each face-to-face contact where the full SDQ was not used	–
	The Youth Top Problems (YTP; Weisz et al., 2011) is an idiographic measure that identifies, prioritises and scores adolescents' three main problems. Each of the nominated problems is scored from 0 (not at all) to 10 (very much), reflecting the extent to which it is a current concern. A mean score is calculated across the nominated problems. The measure has been validated in US clinical populations, where it shows strong evidence of test–retest reliability, convergent and discriminant validity, and sensitivity to change. It was used in Pilot 1 as a screening and outcome measure, and in Pilot 2 as an outcome measure only.	Counsellor at each face-to-face contact	Researcher at baseline/end of intervention
	The Session Feedback Questionnaire (SFQ; Law & Wolpert, 2014) is an ultra-brief 4-item self-report measure of therapeutic alliance, which uses a 5-point Likert scale to assess (i) relational bond between the counsellor and young person, (ii) agreement on session topics, (iii) understanding of session content, and (iv) utility of session content. It is widely used in clinical practice with adolescents in the UK, and also has the advantage of being freely available (unlike similar measures which are only available under paid license). It was used in Pilot 1 to assess the quality of therapeutic alliance over time.	Counsellor at each face-to-face contact	–
Process indicators	An 8-item self-report measure of service satisfaction (Larsen, Attkisson, Hargreaves & Nguyen, 1979) was used to obtain a summative index of intervention acceptability. Total scores range from 8 to 32 (higher scores = greater satisfaction). An established 4-level categorisation system (Smith et al., 2014) was used to benchmark different levels of satisfaction: poor (8–13), fair (14–19), good (20–25) and excellent (26–32).	Researcher at end of intervention	–
	Additional acceptability indicators were derived from referral logs and clinical case records. These were operationalised in terms of demand (numbers and proportions of referred adolescents by referral source/age/grade/gender); uptake (proportion of eligible adolescents participating in at least one session); intervention completion (as a proportion of adolescents starting the intervention), and reasons for non-completion; session attendance (as a proportion of all scheduled sessions); use of materials at home/in sessions, and factors affecting use. Feasibility indicators for intervention delivery were operationalised in terms of number/duration of sessions and length of the completed intervention.	Counsellor (routinely maintained)	Counsellor (routinely maintained)
Qualitative interviews	Individual exit interviews with adolescents were based on a semi-structured topic guide. This examined valued aspects of the intervention; barriers and facilitators to intervention delivery and engagement; and positive and negative outcomes. N = 21 adolescents were purposively sampled to ensure representation across schools, grades and gender.	Researcher, 1–2 weeks after end of intervention	–
	A focus group discussion with counsellors examined the same domains as the adolescent exit interview, with an additional focus on suggested modifications to the intervention. New Delhi counsellors (n = 3) participated alongside other providers with experience of delivering the intervention in Goa (n = 4). Data were recorded using detailed process notes; these were circulated among intervention team members to provide further annotations.	Researcher, mid-way through study	–

### 2.4 Analysis

Formative stage. Sources were synthesised using narrative, thematic and mapping techniques (Chorpita, Bernstein, & Daleiden, 2011; Grant & Booth, 2009; Vaismoradi, Turunen, & Bondas, 2013). Detailed descriptions of the stakeholder analysis and relevance mapping will be described in separate reports and are available on request. Findings were triangulated and combined within a matrix (updated fortnightly) using a constant comparative method (Gale, Heath, Cameron, Rashid, & Redwood, 2013). The matrix addressed an evolving number and variety of formative questions related to intervention design principles and parameters, leading to an intervention ‘blueprint’ based around standardised descriptors (Hoffmann et al., 2014). This iterative and recursive process was led by an Intervention Working Group (DM, KM, MK, RS, AJ & MB) with oversight from senior investigators (VP, CF, BC & PM) and an independent Scientific Advisory Group (see Acknowledgements).

Pilot stage. Quantitative process indicators were described using frequencies, means, SDs and proportions. Analysis of clinical outcome measures involved comparisons of pre-post scores using paired t-tests and was restricted to participants who completed baseline and end-point assessments. In Pilot 1, the post-test score corresponded to the timing of the final intervention session (M = 79.47 days; SD = 19.88). In Pilot 2, post-test scores were collected uniformly at 6 weeks after the pre-test for all participants who were enrolled (M = 45.09 days; SD = 8.86). Effect sizes were calculated as Cohen's d. Due to the small sample size and low power, emphasis was placed on confidence intervals (95% CIs) of effect estimates rather than significance testing. Remission rates were calculated using the ‘crossing clinical threshold’ method (Wolpert et al., 2015) applied to baseline case criteria.

Qualitative data were analysed thematically. All exit interviews were transcribed and analysed in Hindi. To begin, a batch of transcripts were reviewed independently by two coders (RP & KR). Initial deductive codes were derived from research objectives. Additional codes were derived inductively, refined by consensus and ordered into thematic categories conveying inter-related ideas in consultation with the first author (DM). Coders then worked independently to chart text-based fragments from the remaining transcripts into a matrix (codes and categories in columns; individual participants in rows). Regular meetings were used to verify coding decisions and guide further iterations of the framework, comparing and contrasting data within and across interviews. The final framework was also applied to the process notes generated from the focus group with counsellors.

Mixed-methods analysis was used to integrate sources. The main qualitative and quantitative findings were summarised, triangulated and presented together under the main evaluation themes (acceptability, feasibility and impact). Sex-disaggregated analyses were not undertaken owing to relatively small sample sizes in each pilot.

## 3 Formative stage

### 3.1 Results

Formative findings are summarized in Table 1 and the resulting intervention blueprint is summarised in Box 1. An a priori decision was taken to address an array of emotional, behavioural and psychosocial problems using a transdiagnostic intervention architecture. Consistent with this approach, formative interviews revealed a diffuse phenomenology of ‘stress’ and ‘tension’ in adolescents' narratives about mental health and priorities around stress reduction. Multiple referral sources were recommended in intervention design workshops in order to maximise coverage, leading to a plan for targeted sensitisation activities. It was also recommended that eligibility criteria would be operationalised more precisely after reviewing indicators of demand and uptake during initial field testing.

Box 1 **Initial design (‘blueprint’) of a transdiagnostic, low-intensity, psychological intervention for common adolescent mental health problems in Indian secondary schools**

**Eligibility criteria**.

• Wide age range (11–19 years) spanning middle, high and higher secondary school classes
• Referrals to be accepted from teachers, parents and self-initiated routes
• No specific mental health inclusion or exclusion criteria, but cases deemed ‘high risk’ (e.g. due to suicidality) were referred externally to a mental health specialist
• Referrals primarily related to learning difficulties to be excluded

**Theoretical components**.

• Provisional theory of change based on stress-coping principles

**Content/delivery**.

• Problem-focused coping to be addressed using a guided self-help modality, delivered through an illustrated workbook with character-based vignettes
• Emotion-focused coping skills introduced in supplementary handouts matched to problem type(s); intended to provide concrete advice about managing common stress reactions and triggers
• Self-help materials to be supported by counsellor guidance delivered through face-to-face contacts
• Parents would not ordinarily be involved in sessions

**Providers**.

• Lay counsellors to offer brief guidance
• Scalable supervision methods (e.g. peer-led formats) to be emphasised
• Training to address non-specific relational aspects of intervention delivery as well as concepts of problem solving

**Dosing**.

• Delivered over 4 weekly sessions, with an extended initial session to develop a shared understanding of main problems/priorities and to introduce problem-solving concepts
• Brief weekly guidance to support use of self-help materials

**Methods for tailoring**.

• Integrated measurement feedback system to guide intervention planning, including measures of problem/symptom severity, impact and therapeutic alliance
• Targeted handouts for different problem types as a supplement to the main workbook
• Allocation to Step 2 based on simple remission criteria at end of Step 1

Alt-text: Box 1

Another early decision concerned the use of self-help materials supported by non-specialist ‘lay’ counsellors. The global literature has shown that ‘guided’ self-help is more engaging and effective than purely self-directed interventions (Newman, Szkodny, Llera, & Przeworski, 2011), and may be equally effective as face-to-face psychotherapy for common mental health problems in adults (Cuijpers, Donker, van Straten, Li, & Andersson, 2010). Turning to children and adolescents, a recent meta-analysis found that self-help (combining all formats together) was only slightly less effective than face-to-face psychotherapy for common mental health problems, with a magnitude of difference that may not be of clinical significance (Bennett, Cuijpers, Ebert, McKenzie Smith, Coughtrey et al., 2019). In terms of acceptability, self-hep may offer a good fit with adolescents' drive towards independence (Rickwood & Bradford, 2012). Stakeholder interviews endorsed a blended approach involving face-to-face guidance to clarify information and provide corrective feedback while using printed self-help materials. Alternative digital delivery formats appealed to adolescents but were ultimately ruled out due to limited personal ownership of digital devices and acceptability concerns from teachers and parents.

Intervention content was formulated around stress-coping theory (Lazarus & Folkman, 1984), with a technical focus on problem-solving skills to modify and buffer developmentally salient stressors. The fit between problem solving and presenting difficulties in our target population was verified through a relevance mapping exercise based on self-reported problems from a service reference sample of 88 help-seeking adolescents. Problem solving emerged as the most generalizable (i.e., transdiagnostic) element across the range of presenting problems, reflecting its frequent appearance in evidence-based protocols for both externalizing and internalizing difficulties (Boustani et al., 2015; Chorpita & Daleiden, 2009). It was also notable that many reported problems in the sample were indicative of early-stage subthreshold presentations and time-limited adjustment difficulties, for which problem solving may be especially suitable (Cosci & Fava, 2013). To support adolescents seeking concrete advice on coping with age-appropriate challenges (e.g. managing academic demands), it was decided that supplementary handouts would describe relevant situation-specific and emotion-focused coping strategies (e.g. study skills and relaxation).

A further key decision involved situating the guided self-help modality within a larger stepped care architecture. Stepped care models offer the potential to increase acceptability and efficiency of evidence-based health care, by reserving more resource-intensive treatments for individuals who do not benefit from low-intensity first-line interventions (Bower & Gilbody, 2005). Another important feature of stepped care models is their use of ‘self-correcting’ feedback systems, which have also been advocated to improve aspects of shared decision-making in mental health care more generally (Hamilton & Bickman, 2008). Local experts contributed to the identification of a portfolio of idiographic and standardised measures for monitoring progress within our low-intensity Step 1 intervention, and which would ultimately guide decisions about transitioning to a Step 2 treatment of incremental intensity. Some of these measures served an additional purpose as summative evaluative tools in the pilot studies (Table 2).

## 4 Pilot 1

### 4.1 Pre-piloting and intervention modifications

Three postgraduate bilingual psychologists (KM, MK & RS) acted as therapists (one per school) with the intention that they would eventually take on roles as trainers and supervisors to non-specialist counsellors in Pilot 2. The psychologists also shared their experiential learning directly with the Intervention Working Group. Early prototypes of the English-language intervention manual, Hindi-language workbook and supplementary handouts were developed from the blueprint and then field tested during a ‘pre-pilot’ embedding period. Over 600 referrals (mostly for academic problems) were logged in a single month (January–February 2017), likely a reflection of the timing close to year-end exams. Modified eligibility criteria, sensitisation activities and new screening procedures were subsequently developed to target the intervention more efficiently. Difficulties with maintaining session attendance led to a plan for more proactive engagement activities. Modifications were also made to simplify the language and enhance the quality of graphic design in the Step 1 workbook (built around a five-step problem-solving heuristic using the acronym ‘SONGS’) and handouts (Table 3). These modifications were incorporated into an optimised manual and materials for delivery in Pilot 1.

**Table 3:** Evolution of the transdiagnostic, low-intensity, psychological intervention for common adolescent mental health problems in Indian secondary schools.

Intervention parameter	Modifications for Pilot 1	Modifications for Pilot 2
Eligibility criteria	• More narrowly defined age and clinical criteria, assessed by brief standardised tools: (i) enrolled in grades 9–12; (ii) proficient in written/spoken Hindi; (iii) referral was not primarily for a learning difficulty; and (iv) clinically elevated presentation indicated by YTP item score ≥6 or SDQ Impact score ≥2. • Handouts (see below) distributed to students falling below these thresholds.	• Criteria (i) to (iii) were retained. • Criterion (iv) modified as follows: clinically elevated presentation indicated by SDQ Total Difficulties score in Borderline/Abnormal range (≥19 boys, ≥20 girls); SDQ Impact score ≥2; SDQ chronicity item >1 month.
Theoretical components	• Unchanged from blueprint (stress-coping principles).	• Unchanged.
Content/delivery	• Problem solving was the main practice element, delivered through guided self-help. • Printed self-help materials substantially re-designed, with more attractive, colourful illustrations and professional design; shorter and simpler text. • Problem-solving steps presented using the acronym ‘SONGS’: identify a problem situation (S); identify options (O) to solve the problem; narrow down the options by considering pros and cons (N); go for it by trying out the best option (G); sit back and evaluate the outcome (S). • Workbook: new structure (‘learn it, practice it, do it’) applied across each step of problem solving to encourage learning and generalisation from workbook exercises; more varied, realistic vignettes. • Handouts: updated set of 13 handouts structured around SONGS to facilitate integration with workbook; topics included study skills, relaxation, effective communication, stress management, anger management, bullying, understanding love, sexuality, domestic violence, eating healthy, sleep hygiene, making a career choice and managing grief.	• Problem solving retained as main practice element, but delivered through active, counsellor-led face-to-face intervention. • Problem-solving steps presented using the acronym ‘POD’: identify and prioritise distressing/impairing problems (‘Problem identification’); generate and select coping options to modify the identified problem directly (problem-focused strategies), and/or to modify the associated stress response (emotion-focused strategies) (‘Option generation’); implement and evaluate the outcome of this strategy (‘Do it’). • Three psychoeducational ‘POD booklets’ explained problem solving through illustrated stories in comic book format. • Each booklet described a different problem-solving step and suggested corresponding practice exercise; these were distributed sequentially to reinforce learning from sessions and encourage skills practice. • Emotion-focused coping strategies presented as potential options in ‘quick tips’ section of booklets; tips were selected from the most commonly used handouts in Pilot 1 and were no longer matched to presentations. • At the final session, participants received a full-colour POD poster that summarised the three steps of problem solving.
Providers	• Therapists: three (one per school) female psychologists with postgraduate degrees; deployed with the intention that non-specialists would take over at a later stage of piloting. • Three counselling assistants recruited to help with sensitisation, processing of referrals and issuing session reminders. • Supervision structure initially expert-led, with peer group supervision taking up increasing share of the weekly 3-hour allocation.	• Therapists: newly recruited counsellors, including nine college graduates (both males and females) aged above 18 years with no prior training in psychotherapy. • Attended weekly 2-hour peer group supervision meetings, in which they discussed one or two audio-recorded sessions and rated session quality using a structured scale. • Weekly telephone calls (up to 30 minutes) with supervisors (psychologists from Pilot 1) to monitor caseload and manage risk; option for *ad hoc* calls as needed. • Counsellors were also responsible for co-facilitating classroom sensitisation activities with a researcher. • Counsellors received separate manuals for delivering the problem-solving intervention and sensitisation session.
Dosing	• Standard duration of Step 1 extended to 6 weeks, with proactive efforts to schedule face-to-face guidance sessions at weeks 1, 2, 4 and 6. • Flexibility around 2 additional meetings (up to a maximum of 6), according to student need and preference.	• Rapid delivery schedule with 4–5 sessions (20-30-minute duration) delivered over 3–4 weeks. • Flexibility around exact number and spacing of sessions, but emphasis placed on ‘front-loading’ contacts in order to build therapeutic momentum.
Methods for tailoring	• Idiographic problem measure (YTP) used as a method for selecting relevant handouts at intake (also part of eligibility screening). • Session-by-session YTP ratings shared in graphical format and used as basis for collaborative discussions about need for additional guidance sessions.	• Progress assessed using simplified mood and problem measures, incorporating ‘emojis’ on a 5-point Likert scale. • As before, ratings were tracked and reviewed at each session in a graphical format and informed intervention schedule and supervisory discussions.
Sensitisation plan	• Classroom sessions offered a ‘taster’ of problem solving (focused on academic stress) in order to: (i) satisfy demand among students with more transient problems; (ii) socialise students to problem solving; and (iii) provide clear information to students about methods and intended outcomes of school counselling. • Interested students approached the psychologist directly to initiate a referral. • Whole-school sensitisation activities included briefings with school principals and teachers in order to: (i) focus referrals on clinically elevated presentations; and (ii) encourage teachers to discuss referrals with students before passing on details.	• Re-designed classroom sessions emphasised self-identification and normalisation of mental health problems. • Structured around animated video which provided age-appropriate information about types, causes, impacts and ways of coping with common mental health problems, followed by guided group discussion. • Students received a self-referral form with normalising information and question-based prompts to assist with self-identification of mental health problems. • Self-referral could be initiated in person, via the self-referral form, or by depositing a slip with the student's name into a drop-box. • Whole-school sensitisation involved more structured/scripted briefings for school staff.

### 4.2 Participants

**Fig. 2:**
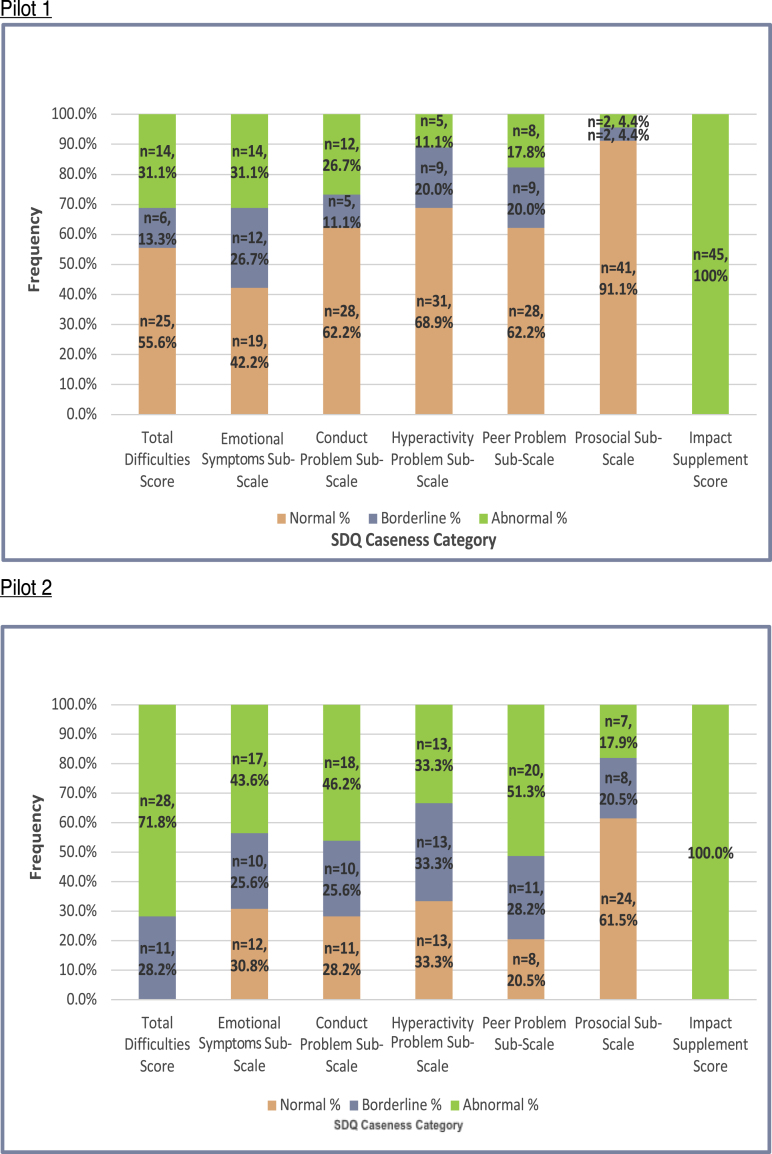
Case characteristics of pilot study participants.

Information about the PRIDE counselling service was disseminated through classroom sessions and whole-school sensitisation activities (Table 3). Adolescents were encouraged to self-refer by directly approaching the school's allocated psychologist. Teachers were advised to speak with adolescents prior to making referrals. Eligibility thresholds (Table 3) were calibrated in pre-pilot work and selected to optimise clinical utility and ecological validity. Ineligible students received one or more handouts corresponding to their identified problem(s). The final sample is described in Table 4, with case characteristics (based on SDQ cut-off scores) illustrated in Fig. 2. Qualitative exit interviews were completed with a sub-sample of 9 females and 12 males (M age = 15.15 years; SD = 1.73).

**Table 4:** Baseline characteristics of pilot study participants.

	Pilot 1 (N = 45)	Pilot 2 (N = 39)
Gender	Female: n = 14 (31.1%) Male: n = 31 (68.9%)	Female: n = 13 (33.3%) Male: n = 26 (66.7%)
Age	M = 15.77 years (SD = 1.77)	M = 15.17 years (SD = 1.16)
Grade	Grade 9: n = 22 (48.9%) Grade 10: n = 3 (6.7%) Grade 11: n = 12 (26.7%) Grade 12: n = 8 (17.8%)	Grade 9: n = 30 (76.9%) Grade 10: n = 5 (12.8%) Grade 11: n = 4 (10.3%) Grade 12: n = 0
Referral source	Self-referral: n = 43 (95.6%) Teacher referral: n = 1 (2.2%) Others (sibling): n = 1 (2.2%)	Self-referral: n = 37 (94.9%) Teacher referral: n = 2 (5.1%) Others: n = 0
SDQ Total Difficulties score	M = 17.53 (SD = 5.65)	M = 23.26 (SD = 3.19)
SDQ Impact score	M = 4.04 (SD = 1.71)	M = 5.21 (SD = 2.47)
YTP score	M = 7.37 (SD = 1.47)	M = 5.50 (SD = 2.66)
SDQ Chronicity	<1 month: n = 0 1–5 months: n = 11 (24.4%) 6–12 months: n = 1 (2.2%) >1 year: n = 33 (73.3%)	<1 month: n = 0 1–5 months: n = 10 (25.6%) 6–12 months: n = 5 (12.8%) >1 year: n = 24 (61.5%)

### 4.3 Results

#### 4.3.1 Acceptability

*Demand and uptake.* N = 175 referrals were received from July–October 2017, accounting for 6.8% of the total student population in 39 sensitised classes. Over half of the referrals (n = 98; 56.0%) were male, consistent with the wider sampling frame. Except for four teacher referrals and one referral initiated by a sibling (also a student at the same school), nearly all referrals (97.1%) were initiated by the index adolescent. Around half of the referrals (n = 91; 52.0%) came from grade 9.

The school-based psychologists screened 108 adolescents (61.7%) in the study period. The remaining referred adolescents (n = 67; 38.3%) opted out (e.g., because they had changed their mind), or else were absent from school for an extended period. After screening, 63 adolescents (58.3%) met study eligibility criteria, from which 45 (71.4%) were enrolled into the study and attended at least one intervention session. The most common reason for non-enrolment was lack of caseload capacity (n = 12). When caseloads were full, eligible adolescents were placed on a waiting list and offered the intervention after a delay but did not participate in the associated research.

Exit interviews with adolescents, conducted by independent researchers, suggested a degree of ambivalence and even worry about accessing counselling, particularly related to concerns about confidentiality and uncertainty about what counselling might involve. Adolescents valued clear and up-front assurances about privacy during sensitisation and screening activities, as well as hopeful messages and friendly interactions with counsellors during the same. The intervention providers expressed concerns that teachers were generally sceptical and disengaged from the referral process, leading to suggestions for more focused teacher sensitisation activities. Instances were also reported of female psychologists experiencing verbal harassment from male teaching staff and students, leading to the recommendation that male counsellors should be deployed in all-boys schools.

*Engagement with guidance sessions and self-help materials.* Thirty-eight adolescents (84.4%) completed the intervention, defined as attendance at 75% or more of scheduled guidance sessions, sustained over six weeks. Six adolescents dropped out and did not provide reasons; one adolescent explained that their problem had improved.

Use of the self-help workbook was highly variable and only six students completed all 15 sections by the end of the intervention (M = 8.5 sections; SD = 4.2; range 0–15). Non-completion of the workbook between sessions was documented at least once for most adolescents (n = 36). Remedial workbook completion took place within sessions, but was constrained by the fact that adolescents failed to bring their workbook to 61.1% of follow-up meetings; eight adolescents (17.8%) did not bring their workbook to a single session. The most commonly distributed handouts (tailored to specific adolescent presentations or concerns) covered study skills (n = 20; 44.4%), stress management (n = 17; 37.8%), effective communication (n = 16; 35.6%), relaxation (n = 11; 24.4%) and anger management (n = 9; 20.0%).

Analysis of clinical case records, corroborated by exit interviews, revealed that the most common barriers to engaging with the workbook were difficulties with or lack of interest in reading/writing, lack of retained knowledge/conceptual understanding about problem solving, and insufficient time due to exams/other academic commitments. Positive responses about the workbook emphasised the relatability of character-based narrative vignettes to students’ interests and personal circumstances. Adolescents appreciated the brief handout format and topic-specific coping tips, but literacy and time concerns were also noted as barriers to use.

Face-to-face sessions with the psychologist helped to compensate for many of the perceived challenges of the self-help approach. In particular, adolescents valued the practical/facilitative role of the psychologist in providing corrective feedback and encouragement on completed workbook exercises, explaining difficult concepts and words, and generating specific solutions to problems. More significantly, most adolescents considered the quality of the therapeutic relationship to be of central importance to their engagement and outcomes in counselling. Indeed, for several participants, the problem-solving content was judged to be merely incidental compared to the potent relational ingredient of the guidance sessions. In-session YTP assessments (along with graphical representations of previous ratings) were also viewed favourably by adolescents, as a way to highlight therapeutic gains and reinforce coping efforts. Other questionnaires were considered more difficult to understand and sometimes caused confusion in sessions. Parental involvement was minimal in practice, and there was little interest in revisiting this option.

Intervention providers mirrored adolescents' views about barriers and facilitators to using self-help materials, with suggestions made for increasing graphical content, and further simplifying the problem-solving heuristic and accompanying text. Other suggestions concerned the use of a consolidated Likert scale to streamline in-session assessments. Providers additionally reflected on the apparent mismatch between a required therapeutic stance involving supported autonomy, and a culturally sanctioned ‘teacher-student’ model based on ‘giving the right answer.’ More explicit attention to the therapeutic relationship was suggested to resolve this tension.

*Service satisfaction.* Mean service satisfaction scores ranged from good to excellent (M = 28.55; SD = 2.48; range = 22–32). All 38 respondents felt that the service had helped them to deal more effectively with their problems and would recommend counselling. However, seven participants (18.4%) were dissatisfied with the amount of help they received, and expected more.

#### 4.3.2 Feasibility

The intervention manual allowed for up to six sessions over six weeks, but none of the intervention completers attended more than five sessions (M = 3.82 sessions; SD = 0.73; range = 3–5). In practice, however, the total length of the completed intervention typically extended beyond the 6-week target (M = 52.45 days; SD = 13.66; range = 27–83). Individual sessions were brief relative to the allotted 30-minute class period (M = 23.56 minutes; SD = 5.24; range 17–37 ), with around half of this time used for ‘guidance’ (M = 12.61 minutes; SD = 3.97; range 7–24 ) and the balance used for progress monitoring. There was consensus among interviewed adolescents that an optimal schedule would involve 20–30 minutes per session and around four sessions in total, closely mirroring the observed pattern. Providers endorsed the importance of a brief delivery schedule to maintain feasibility (and acceptability), recommending a reduction in assessment procedures to enable proportionately more guidance/therapeutic content in each meeting.

#### 4.3.3 Impact

Clinical outcomes for intervention completers (n = 38) are summarised in Table 5. Moderate to very large effects were found in the intended direction on the SDQ Total Difficulties score (d = 0.79; 95% CI = 0.42–1.15), Impact score (d = 1.99; 95% CI = 1.43–2.54) and YTP (d = 1.89; 95% CI = 1.35–2.42), indicating potential for generalised effects across the totality of presenting problems/symptoms and also on associated distress/impairment. Session-by-session assessments showed consistent upward trajectories on the SFQ (increasing therapeutic alliance) and downward trajectories on the YTP and SDQ SxS (decreasing problems and associated impact); graphical summaries are available on request. Almost three-quarters of participants were fully remitted by the final assessment point (with idiographic problem and impact scores both dropping below eligibility thresholds); only one adolescent (2.6%) failed to respond on any criteria.

**Table 5:** Clinical outcomes.

	Pilot 1 (original eligibility criteria; N = 38)^a^	Pilot 1 (sub-analysis based on Pilot 2 eligibility criteria; N = 16)	Pilot 2 (N = 29)^b^
SDQ Total Difficulties	Pre: M = 17.53 (SD = 5.66) Post: M = 13.32 (SD = 5.64) t(37) = 4.87 (p < 0.001) **d = 0.79 (95% CI = 0.42**–**1.15)**	Pre: M = 22.75 (SD = 2.77) Post: M = 15.56 (SD = 7.21) t(15) = 4.61 (p < 0.001) **d = 1.15 (95% CI = 0.50**–**1.78)**	Pre: M = 22.79 (SD = 2.97) Post: M = 15.93 (SD = 6.14) t(28) = 6.93 (p < 0.001) **d = 1.29 (95% CI = 0.79**–**1.78)**
SDQ Emotional Problems sub-scale	Pre: M = 5.71 (SD = 2.31) Post: M = 4.21 (SD = 2.34) t(37) = 4.11 (p < 0.001) **d = 0.67 (95% CI = 0.31**–**1.02)**	Pre: M = 7.31 (SD = 1.25) Post: M = 5.31 (SD = 2.80) t(15) = 3.76 (p = 0.002) **d = 0.94 (95% CI = 0.34**–**1.52)**	Pre: M = 6.50 (SD = 1.99) Post: M = 4.50 (SD = 2.49) t(28) = 3.46 (p = 0.002) **d = 0.64 (95% CI = 0.24**–**1.04)**
SDQ Conduct Problems sub-scale	Pre: M = 3.74 (SD = 2.06) Post: M = 2.34 (SD = 1.88) t(37) = 4.12 (p < 0.001) **d = 0.67 (95% CI = 0.31**–**1.02)**	Pre: M = 5.06 (SD = 1.57) Post: M = 2.63 (SD = 2.22) t(15) = 4.07 (p = 0.001) **d = 1.02 (95% CI = 0.40**–**1.62)**	Pre: M = 4.57 (SD = 1.71) Post: M = 2.96 (SD = 1.88) t(28) = 3.93 (p = 0.001) **d = 0.73 (95% CI = 0.31**–**1.14)**
SDQ Hyperactivity sub-scale	Pre: M = 4.39 (SD = 1.90) Post: M = 4.29 (SD = 1.71) t(37) = 0.29 (p = 0.770) **d = 0.05 (95% CI = -0.27**–**0.36)**	Pre: M = 5.69 (SD = 1.35) Post: M = 4.63 (SD = 2.09) t(15) = 1.95 (p = 0.070) **d = 0.49 (95% CI = -0.04**–**1.00)**	Pre: M = 5.96 (SD = 1.86) Post: M = 4.43 (SD = 1.79) t(28) = 4.81 (p < 0.001) **d = 0.89 (95% CI = 0.46**–**1.32)**
SDQ Peer Problems sub-scale	Pre: M = 3.68 (SD = 1.80) Post: M = 2.47 (SD = 1.39) t(37) = 4.17 (p < 0.001) **d = 0.68 (95% CI = 0.32**–**1.03)**	Pre: M = 4.69 (SD = 1.35) Post: M = 3.00 (SD = 1.46) t(15) = 3.72 (p = 0.002) **d = 0.93 (95% CI = 0.33**–**1.51)**	Pre: M = 5.75 (SD = 1.65) Post: M = 4.04 (SD = 1.57) t(28) = 6.00 (p < 0.001) **d = 1.11 (95% CI = 0.64**–**1.57)**
SDQ Impact	Pre: M = 4.11 (SD = 1.77) Post: M = 0.32 (SD = 0.93) t(37) = 12.26 (p < 0.001) **d = 1.99 (95% CI = 1.43**–**2.54)**	Pre: M = 4.50 (SD = 2.16) Post: M = 0.56 (SD = 1.31) t(15) = 6.78 (p < 0.001) **d = 1.70 (95% CI = 0.91**–**2.46)**	Pre: M = 4.89 (SD = 2.33) Post: M = 1.68 (SD = 2.29) t(28) = 6.30 (p < 0.001) **d = 1.17 (95% CI = 0.69**–**1.64)**
YTP	Pre: M = 7.18 (SD = 1.45) Post: M = 2.62 (SD = 1.89) t(37) = 11.65 (p < 0.001) **d = 1.89 (95% CI = 1.35**–**2.42)**	Pre: M = 7.49 (SD = 1.78) Post: M = 3.03 (SD = 1.95) t(15) = 6.48 (p < 0.001) **d = 1.62 (95% CI = 0.85**–**2.36)**	Pre: M = 5.41 (SD = 2.76) Post: M = 2.71 (SD = 2.67) t(28) = 4.88 (p < 0.001) **d = 0.91 (95% CI = 0.47**–**1.33)**
Remission rate -Full -Partial -None	**n = 28 (73.7%)** n = 9 (23.7%) n = 1 (2.6%)	**n = 8 (50.0%)** n = 8 (50%) n = 0 (0.0%)	**n = 13 (44.8%)** n = 10 (34.5%) n = 6 (20.7%)

a Non-completers (n = 7) tended to be older (M = 15.54 years vs 17.06 years) and have higher YTP scores at baseline (M = 8.40 vs 7.18), but did not differ significantly (p > 0.05) from intervention completers on the basis of sex or SDQ scores.

b Non-completers (n = 10) did not differ significantly (p > 0.05) from intervention completers on the basis of age, sex or baseline scores.

Adolescent interviews reiterated the observed changes in symptoms (reduced ‘tension’ and anger being especially common) and functional impacts (related especially to family/peer relationships and academic performance). In terms of negative outcomes, a minority of students pointed to dismissive attitudes from peers, teachers and family members about the value of counselling. However, most participants explicitly denied stigma around counselling. They commonly described curiosity (even envy) from peers and siblings, with plentiful examples of workbooks and handouts being shared, copied and borrowed.

## 5 Pilot 2

### 5.1 Pre-piloting and intervention modifications

Pilot 1 findings were reviewed in detail by the Intervention Working Group and members of the Scientific Advisory Group in late 2017, leading to a series of important modifications (Table 3). First, new eligibility criteria were formulated to manage demand and ensure that more highly symptomatic, impaired and chronic cases are selected for Step 1. Additional classroom-based sensitisation activities, including a new psychoeducational video (https://youtu.be/NyWahyiFk9c), were developed to generate proportionately more clinically elevated referrals. Students were also able to self-refer by leaving their details in a secure ‘drop box,’ rather than having to approach an adult gatekeeper directly. Second, the style of Step 1 delivery was modified in line with adolescents' expectations and preferences for a more active therapeutic stance from counsellors (i.e., moving away from a predominantly self-help modality and towards a counsellor-led, low-intensity intervention). Third, the five problem-solving steps used in the original self-help materials were simplified into a three-step heuristic (POD; ‘Problem-Option-Do it’). This was introduced and explained in context- and age-appropriate comic book stories (‘POD booklets’) rather than through written exercises (Supplementary Files). Fourth, to foster engagement and to identify non-responders who might need ‘stepping up’ more quickly, a brief delivery schedule was specified in which four sessions would be delivered over three weeks (front-loaded with two sessions in week one and weekly sessions thereafter). Provision was made for an optional fifth session to enable further practice of problem-solving skills and to consolidate gains. Pre-piloting was used to re-design problem-solving and sensitisation materials, re-draft corresponding manuals, and recruit/train a new cohort of non-specialist counsellors. Details of all modifications for Pilot 2 are presented in Table 3.

### 5.2 Participants

Participants were required to meet updated case criteria, with the idiographic YTP problem score replaced by a standardised assessment of symptom severity using the SDQ Total Difficulties score (selecting cases in the borderline or abnormal range). Participants were additionally required to score in the abnormal range on the SDQ Impact Supplement, with the chronicity of their difficulties lasting for more than 1 month. Other eligibility criteria remained unchanged from Pilot 1. Participant characteristics are shown in Table 4 and Fig. 2.

### 5.3 Results

#### 5.3.1 Acceptability

*Demand and uptake.* N = 326 referrals were logged over the study period from July–August 2018, representing 17.5% of the total student population in the 45 sensitised classes. This included 13 classes receiving a classroom sensitisation session for the first time, and 32 classes that had already received a sensitisation session during the pre-pilot stage. The latter received a ‘reminder visit’ from a counsellor. As before, the vast majority of referrals (n = 316; 96.9%) came directly from adolescents, with the balance made up from teachers (n = 6; 1.8%) and others (n = 4; 1.2%). Referrals were mostly boys (n = 227; 69.6%) and from grade 9 (n = 261; 80.1%), reflecting the distribution of sensitised classes.

Two hundred and seventy-three referrals were screened, although 53 referrals (16.3%) opted out due to literacy difficulties (n = 23), ongoing mental health treatment (n = 4), unavailability (n = 4) and unspecified reasons (n = 22). Sixty-seven (24.5%) of the screened adolescents met study eligibility criteria, of which n = 39 (58.2%) were enrolled and participated in the intervention. Reasons for non-enrolment/non-participation in the intervention were lack of caseload capacity (n = 14) and declined research consent (n = 14). Students in the latter category were offered the intervention after a delay but did not participate in the associated research. For the consenting/waitlisted participants, repeated measures were obtained at baseline and again after a six-week wait, providing a naturalistic control group for which n = 12 were successfully followed up.

*Engagement with sessions and materials.* Twenty-nine of the enrolled adolescents (74.4%) completed the intervention, defined as attendance at four or five sessions. Six adolescents dropped out either because they were not interested or did not provide reasons; two adolescents explained that their problems improved and they no longer needed help; one adolescent said that they no longer had time; and one adolescent was absent from school for an extended period.

Across all 115 follow-up sessions, there were only 13 documented instances (11.3%) in which a student did not complete a suggested ‘homework’ activity before a session, and five sessions (4.3%) where the adolescent did not bring their POD booklet. Comprehension difficulties related to the POD booklet were documented in two sessions, while lack of understanding about problem-solving concepts/skills was noted in seven sessions.

#### 5.3.2 Feasibility

Most intervention completers (n = 20; 69.0%) received the maximum dose (M = 4.90 sessions; SD = 0.31; range 4–5) over a relatively rapid schedule (M = 22.55 days; SD = 6.03; range = 14–34 days). Individual sessions were mostly completed within the standard 30-minute class period (M = 22.98 minutes; SD = 7.16; range = 13–60).

#### 5.3.3 Impact

Clinical outcomes for intervention completers are summarised in Table 5 and benchmarked against Pilot 1 results, including a sub-analysis of Pilot 1 outcomes focused only on participants who would have satisfied Pilot 2's more stringent eligibility criteria. Within the Pilot 2 cohort, pre-post analyses revealed moderate to very large effects on SDQ Total Difficulties, SDQ Impact and YTP scores. Confidence intervals overlapped with effect sizes from the Pilot 1 reference sub-group, although a trend was visible towards relatively stronger effects in Pilot 1 on SDQ Impact and YTP scores. Just under half (44.8%) of Pilot 2 participants were fully remitted at 6 weeks (compared with 50.0% for the Pilot 1 benchmarking sub-group), while six adolescents in Pilot 2 (20.7%) failed to respond on any criteria (compared with none in the Pilot 1 benchmarking sub-group). A *post hoc* between-group analysis was also undertaken to compare intervention completers with a waitlisted control group in Pilot 2. This revealed attenuated effect sizes with wide confidence intervals for the SDQ Total Difficulties score (d = 0.40; 95% CI = −0.20 to 0.98), and for the SDQ Impact score (d = 0.65; 95% CI = 0.01 to 1.26). Effect sizes were even lower for the other outcomes measures, notably including the YTP (d = 0.01; 95% CI = −0.36 to 0.36).

## 6 Discussion

This paper has charted the inception and evolution of a transdiagnostic, low-intensity intervention addressing a wide range of emotional and behavioural problems among adolescents attending Government-run secondary schools in India. The design process spanned two and a half years, applying a systematic methodology that integrated multiple sources of local and global evidence to produce an initial design specification (nested within a wider stepped care architecture), followed by iterative piloting and refinements. Key findings were: (i) the identification of problem solving as the primary mechanistic element of the intervention; (ii) the re-formulation of eligibility criteria and corresponding sensitisation activities to ensure more efficient targeting while minimising the burden of assessment; (iii) a change in therapeutic modality from guided self-help to a more engaging counsellor-led therapy; (iv) an extensive re-design of intervention materials from self-completed workbooks to psychoeducational comic books; and (v) a revised ‘front-loaded’ delivery schedule to build therapeutic momentum, mitigate feasibility challenges around the school timetable/calendar and thereby maximise engagement.

The key implementation processes for the optimised intervention are summarised in Box 2. The relative paucity of such descriptions in previous LMIC-based intervention studies has been highlighted in a recent state-of-the-art review by Singla and colleagues (2017). It is hoped that our systematic methodology and the structured reporting of the emergent intervention specification will stimulate future efforts to develop effective and scalable mental health innovations globally.

Box 2 **Implementation processes1 for optimised version of PRIDE ‘Step 1’ low-intensity intervention.**

**Where?**

Intervention setting: Government-run secondary schools in New Delhi, India but with potential to be rolled out to other locations and in schools run by NGOs and other education providers.

Rationale for the setting: opportunities to reach a large, high-need adolescent population in a context that lacks specialised services for adolescent mental health care.

Notable facilitators: permissions from school authorities; access to classrooms for universal sensitisation activities.

Notable barriers related to the setting: students’ concerns about confidentiality/stigma and literacy difficulties; lack of involvement from parental caregivers; lack of physical infrastructure (e.g. private rooms); gaps in school calendar due to exam breaks and holidays.

**What?**

Intervention class: problem-solving therapy.

Intervention components:

*Nonspecific elements:* collaboration; empathy; active listening; normalisation; eliciting commitment; discussing advantages of the intervention; discussing barriers to engagement.

*Specific elements:* problem solving; self-monitoring; linking affect to life events.

*Other in-session techniques:* assigning homework; reviewing homework; goal setting; psychoeducation; giving direct suggestions; praise by therapist.

Adaptations for specific context or target group: rapid delivery schedule; illustrated booklets.

**Who?**

Delivery agent: non-specialist 'counsellors' with college degrees but no formal training or qualifications related to psychotherapy or mental health.

Selection criteria: recruited through online job portals commonly used in the local NGO/public sector; selection based on reasoning capacity (assessed by written test) and interpersonal skills (assessed by structured role-plays and interview).

Demographics: Hindi-speaking; aged 18 years and above; mixture of males (assigned to all-boys schools) and females.

Notable facilitators related to the choice of delivery agent: abundance of college graduates in local setting; contractual accountability of intervention providers to the PRIDE programme.

Notable barriers related to the choice of delivery agent: harassment of female staff in all-boys schools; perceived overlap with expansion of local Educational and Vocational Guidance Counsellors (EVGCs) has limited scale of implementation in New Delhi, while also creating an opportunity for collaboration with this expanding Government-supported cadre.

Compensation for the delivery agent: salaried employees of implementing organisation (Sangath NGO).

Certification processes: internal processes based on completing all training requirements (see below).

**How?**

Training:

*Trainers:* master's and doctoral-level psychologists with at least 3 years of post-qualification experience.

*Format and duration of training:* one week of classroom-based training involving a combination of lectures, demonstrations and role-plays; followed by a minimum 6-week period of field training during which counsellors carry out casework with at least four cases under supervision; recurrent skills deficits noted by supervisors are addressed through supplementary training workshops held on a monthly basis.

*Procedures for assessing competence:* structured role-plays at the end of classroom-based training and supervisors’ ratings of audio-recorded intervention sessions.

Supervision:

*Supervisors:* psychologists and peers.

*Format and methods of supervision:* weekly 2-hour peer group supervision meetings, facilitated by one of the counsellors in rotation and overseen by a supervising psychologist; counsellors discuss and rate one or two audio-recorded sessions in each group meeting using a structured therapy quality rating scale (see below); weekly 1:1 telephone calls (20–30 minutes each) are offered by supervising psychologists to individual counsellors in order to monitor progress of their caseload, with option for *ad hoc* calls as needed.

Intervention characteristics:

*Delivery format:* individual, face-to-face.

*Duration of intervention:* 3 weeks.

*Number of sessions:* 4–5 sessions delivered in temporal sequence.

*Length of sessions:* 20–25 minutes.

*Quality assessment:* based on a therapy quality rating scale that assesses performance of relevant in-session techniques, using formats consistent with established scales (Kohrt et al., 2015; Muse, McManus, Rakovshik and Thwaites, 2017).

*Materials:* participants receive three colour-printed 'POD booklets' that explain problem solving using illustrated vignettes and describe corresponding home practice exercises; participants also receive a poster in their final session that summarises the three steps of problem solving and is intended to encourage generalisation of skills across contexts; counsellors receive a session-by-session intervention manual, visual aids to illustrate intervention structure and rationale, a progress monitoring tool, and a set of session record forms.

Alt-text: Box 2

^1^ Descriptive framework based on checklist developed by Singla et al. (2017).

In approaching the design of a school-based mental health intervention, we were mindful that the significant potential to screen and treat large numbers must be balanced against students' commonly reported concerns about confidentiality and stigma. The importance of clear messages about privacy emerged strongly in our formative and exit interviews with adolescents, and is well recognised in the global literature on school mental health services (Gronholm, Nye, & Michelson, 2018). Comparing between the two pilot studies, we found that the referral rate (as a proportion of the total sampling frame in sensitised classes) more than doubled from 6.8% to 17.5%. This may be an indication that the modified classroom sensitisation session (involving an animated video) was relatively effective in generating awareness and overcoming other barriers to referral. Although the eligibility rate for screened referrals dropped substantially between the two pilots (from 58.3% in Pilot 1 to 24.5% in Pilot 2), this was expected due to the raising of clinical thresholds. The specific impacts of the sensitisation activities will be explored in further PRIDE research (Parikh et al., 2019). Other important unanswered questions concern the needs and expectancies of help-seeking adolescents who fall below clinical eligibility thresholds for the problem-solving intervention, particularly given that around three-quarters of screened referrals did not meet criteria in Pilot 2. A better understanding of these sub-threshold referrals could guide efforts to develop preventive interventions on a universal or ‘open-access’ basis (i.e., as a ‘Step 0’ in an elaborated stepped care architecture).

In its final variant, the Step 1 problem-solving intervention was delivered by a team of non-specialist counsellors, typically in five 20–25-minute sessions spread across three weeks. The resulting outcomes suggest strong potential for impact, with Pilot 2 results (delivered by non-specialist counsellors) broadly on par with the psychologist-delivered precursor in Pilot 1. The remission rates in Pilot 2 and Pilot 1 (adjusted for Pilot 2 baseline criteria) were 44.8% and 50.0% respectively, within the benchmark of 40–60% typically achieved by evidence-based treatments for common adolescent mental health problems in ‘real-world’ clinical trials from high-income countries (Lee, Horvath, & Hunsley, 2013). Uncontrolled effect sizes on continuous measures were also promising (moderate to very large effects on overall psychopathology, impact and problem scores), although a *post hoc* analysis of Step 2 outcomes compared with a waitlisted control group revealed somewhat smaller (but imprecise) effects with wide confidence intervals. However, we acknowledge that the various outcomes reported in our pilot studies must be viewed as preliminary and interpreted cautiously given the small sample sizes, absence of *a priori* control conditions and lack of long-term follow-up. The latter is especially important given evidence that remitted (adult) participants in low-intensity psychological interventions may show substantial relapse rates within one year (Ali et al., 2017). In addition, the demand characteristics of assessments in Pilot 1 (where psychologists administered baseline measures) may have influenced scores and could possibly explain the trend in the first pilot towards relatively larger effect sizes on some outcome measures. The same trend could also be explained by the more experienced therapists and/or the longer follow-up period in Pilot 1, which may have permitted more time for spontaneous problem resolution.

Notwithstanding these caveats, it is notable that all outcomes were achieved using a delivery schedule that was brief even by the standards of other low-intensity interventions designed for LMICs. For example, the WHO-supported 'Problem Management Plus' – another transdiagnostic intervention with problem-solving as the core element, but targeted to adults – is delivered over five 90-minute weekly sessions (Dawson et al., 2015). We found that a concise schedule increased feasibility in the context of a busy school schedule which required sessions to be integrated within breaks in the class timetable, or within a single class period. In addition, the start of the brief intervention could be calibrated more easily to fit around holidays and exam breaks. There was also a good fit to adolescents’ stated priority for rapid stress reduction and a requirement to limit the time missed from class. The latter consideration was raised by a number of stakeholders at the formative stage and has particular importance in Indian schools, where students sit for frequent examinations and academic pressure is a common contributing factor in mental health presentations (Parikh et al., 2019; Parikh, Sapru, Krishna, Patel, & Michelson, 2019.

These and other insights attest to the value of engaging directly with adolescents at multiple stages of intervention development. Previous research has highlighted that adolescents have distinctive mental health needs and help-seeking preferences that often diverge from younger children and adults (McGorry, 2007). This can result in mismatches with ‘downward adaptations’ of adult protocols or ‘upward adaptations’ of child mental health interventions (Weisz & Hawley, 2002). Although we did not gain traction for the use of guided self-help (which is otherwise well established with adults), our alternative ‘lean’ intervention design is aligned with recent innovations emerging from co-production efforts with young people in high-income countries (Sclare & Michelson, 2016). Additional strengths of our study relate to the triangulation of multiple data sources and the prospective two-stage cohort design. This iterative approach to piloting enabled modifications to be planned and evaluated in quick succession. In addition to the research limitations noted above, we also acknowledge inconsistencies in the data sources that were available across the two pilots, such that more data were available in Pilot 1. On the other hand, experiential learning at the pre-pilot stage mitigated against major gaps in understanding feasibility/acceptability issues.

## 7 Conclusions

Formative and pilot results suggest that the PRIDE ‘Step 1’ intervention has the potential to be a cost-effective first-line transdiagnostic intervention for common adolescent mental health problems in India and other low-resource settings. A subsequent randomised controlled trial will provide a definitive test of its effectiveness, alongside an embedded recruitment trial that will evaluate the specific impacts of sensitisation activities on referral generation (Parikh et al., 2019). Additional studies will evaluate a higher-intensity ‘Step 2’ treatment for persistent presentations. These inter-linked research efforts will shape the final specifications and implementation of a comprehensive stepped care programme that is intended to reduce the adolescent mental health burden at scale, in India and globally.

## Funding

This work was supported by the 10.13039/100010269Wellcome Trust, UK (Grant number 106919/Z/15/Z).

## Conflicts of interest

The authors declare no conflict of interest.
