# Supplementary material for: Development of a transdiagnostic, low-intensity, psychological intervention for common adolescent mental health problems in Indian secondary schools
Source: Behav Res Ther. 2020 Jul;130:103439. doi: 10.1016/j.brat.2019.103439 (PMC7322400; doi:10.1016/j.brat.2019.103439)
Supplement: Multimedia component 1 [file mmc1.pdf]

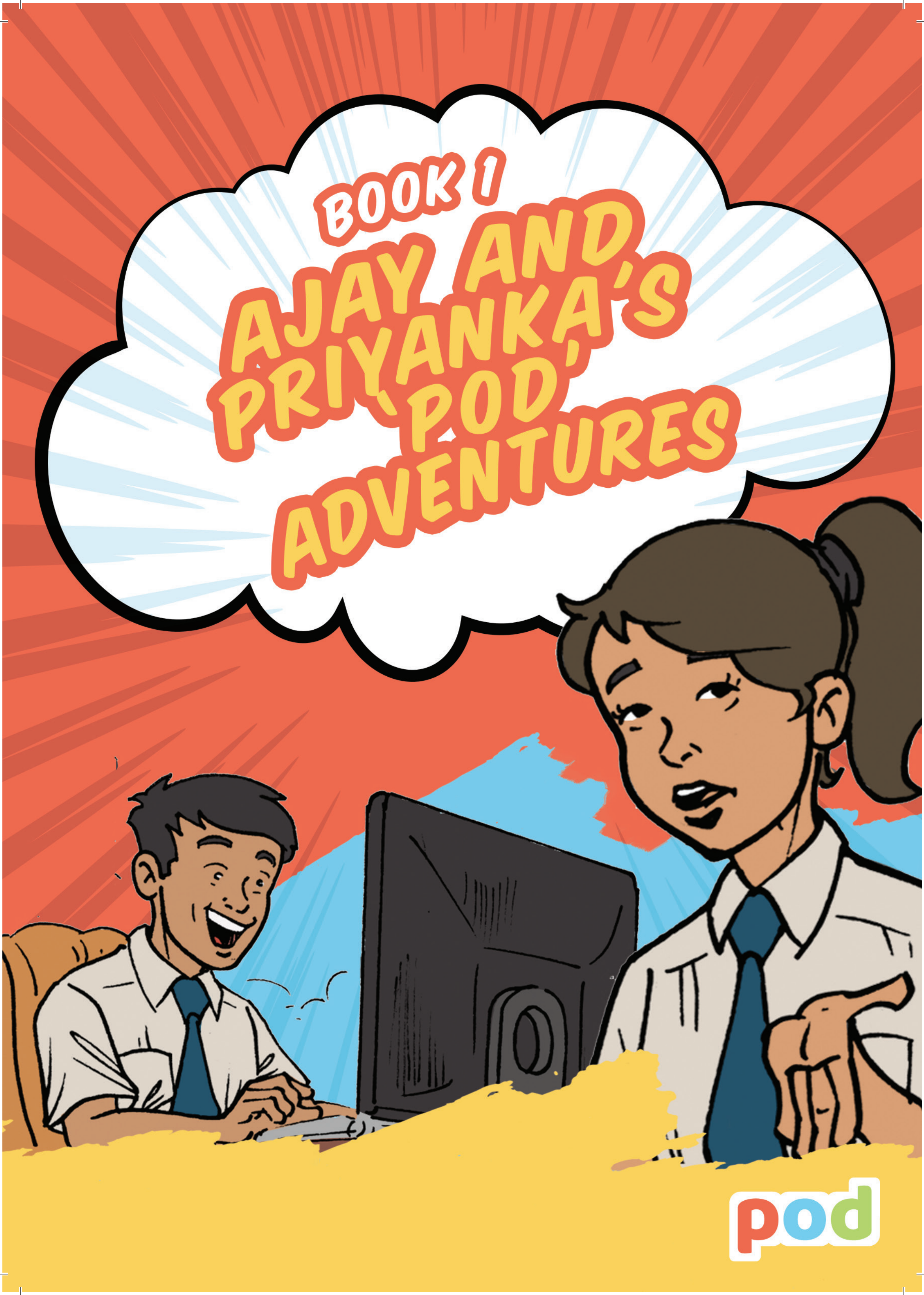
BOOK 1
AJAY AND PRIYANKA'S 'POD' ADVENTURES
pod

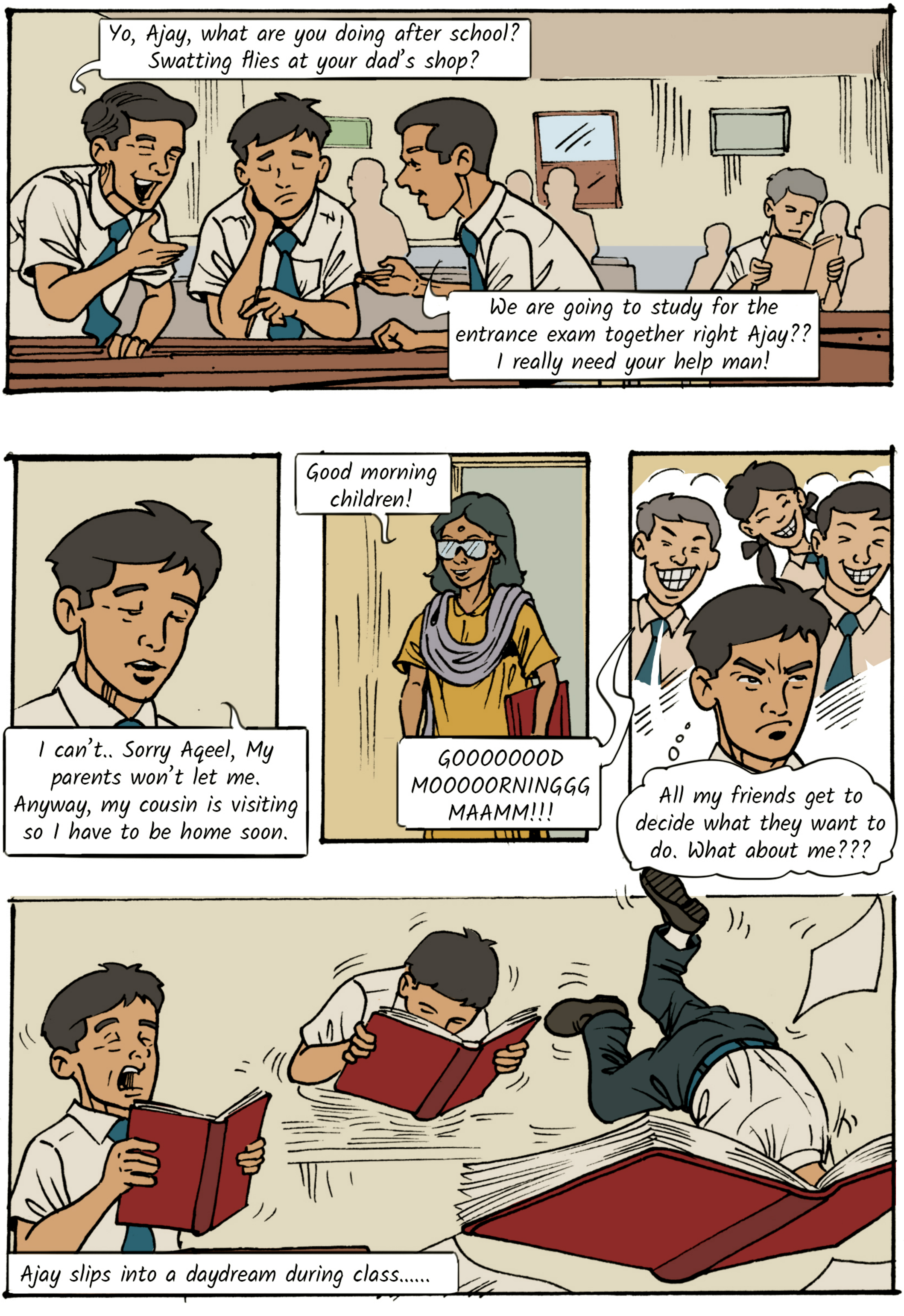
Yo, Ajay, what are you doing after school? Swatting flies at your dad's shop?
We are going to study for the entrance exam together right Ajay?? I really need your help man!
I can't.. Sorry Aqeel, My parents won't let me. Anyway, my cousin is visiting so I have to be home soon.
Good morning children!
GOOOOOOOD MOOOOORNINGGG MAAMM!!!
All my friends get to decide what they want to do. What about me???
Ajay slips into a daydream during class......

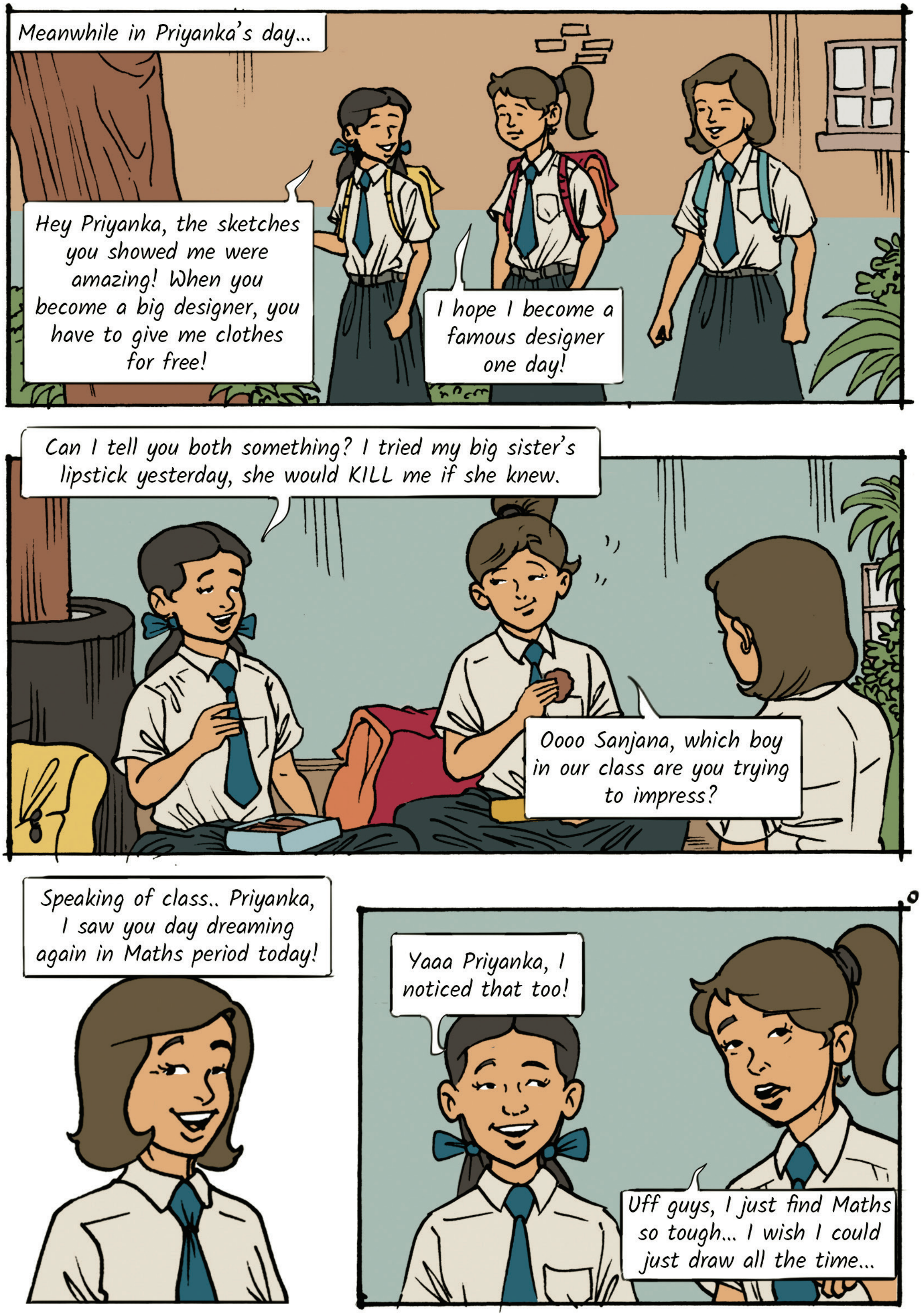
Meanwhile in Priyanka's day...
Hey Priyanka, the sketches you showed me were amazing! When you become a big designer, you have to give me clothes for free!
I hope I become a famous designer one day!
Can I tell you both something? I tried my big sister's lipstick yesterday, she would KILL me if she knew.
Oooo Sanjana, which boy in our class are you trying to impress?
Speaking of class.. Priyanka, I saw you day dreaming again in Maths period today!
Yaaa Priyanka, I noticed that too!
Uff guys, I just find Maths so tough... I wish I could just draw all the time...

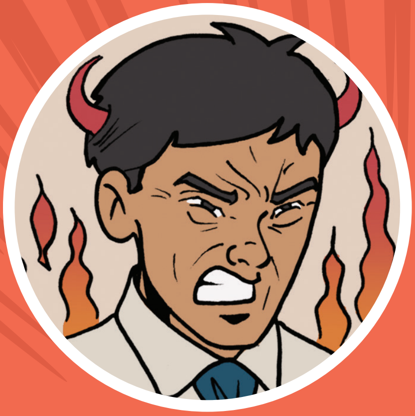

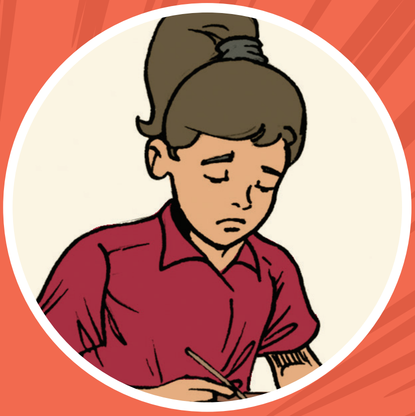

Hope you enjoyed reading about Ajay and Priyanka! Both brother and sister face a few challenges in their life-just like all of us do! What do you think Ajay and Priyanka's biggest problems are? To see how they handle these challenges, read Book 2!

pod

What about you? Take a few moments to think about your life - your family, friends, school and yourself. Are there any difficult life situations you are experiencing? Write down your problems and how they affect you in the blank space ahead - you can list as many as you want!
