# Supplementary material for: Development of a transdiagnostic, low-intensity, psychological intervention for common adolescent mental health problems in Indian secondary schools
Source: Behav Res Ther. 2020 Jul;130:103439. doi: 10.1016/j.brat.2019.103439 (PMC7322400; doi:10.1016/j.brat.2019.103439)
Supplement: Multimedia component 2 [file mmc2.pdf]

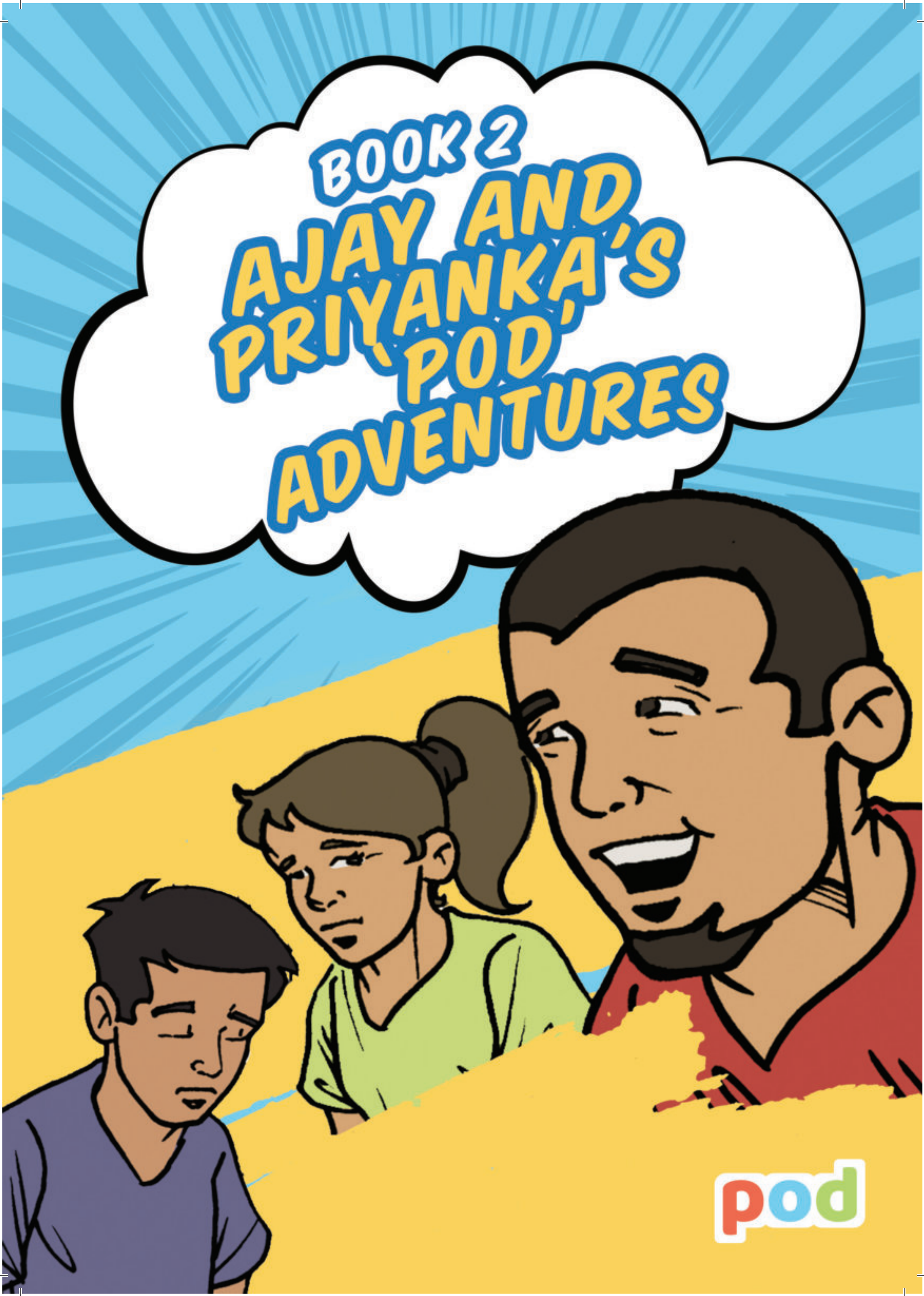
BOOK 2
AJAY AND PRIYANKA'S 'POD' ADVENTURES
pod

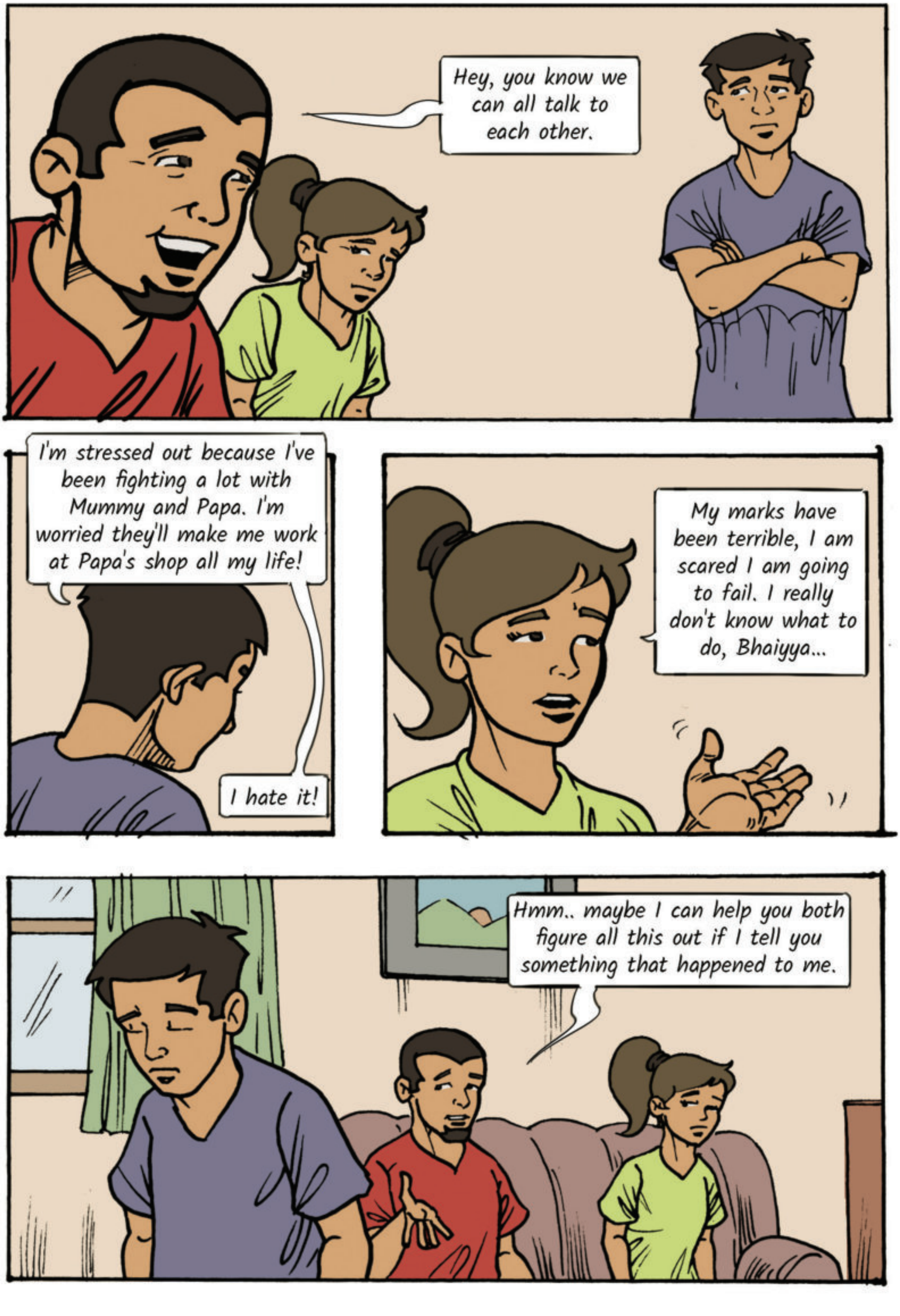
Hey, you know we can all talk to each other.
I'm stressed out because I've been fighting a lot with Mummy and Papa. I'm worried they'll make me work at Papa's shop all my life!
I hate it!
My marks have been terrible, I am scared I am going to fail. I really don't know what to do, Bhaiyya...
Hmm.. maybe I can help you both figure all this out if I tell you something that happened to me.

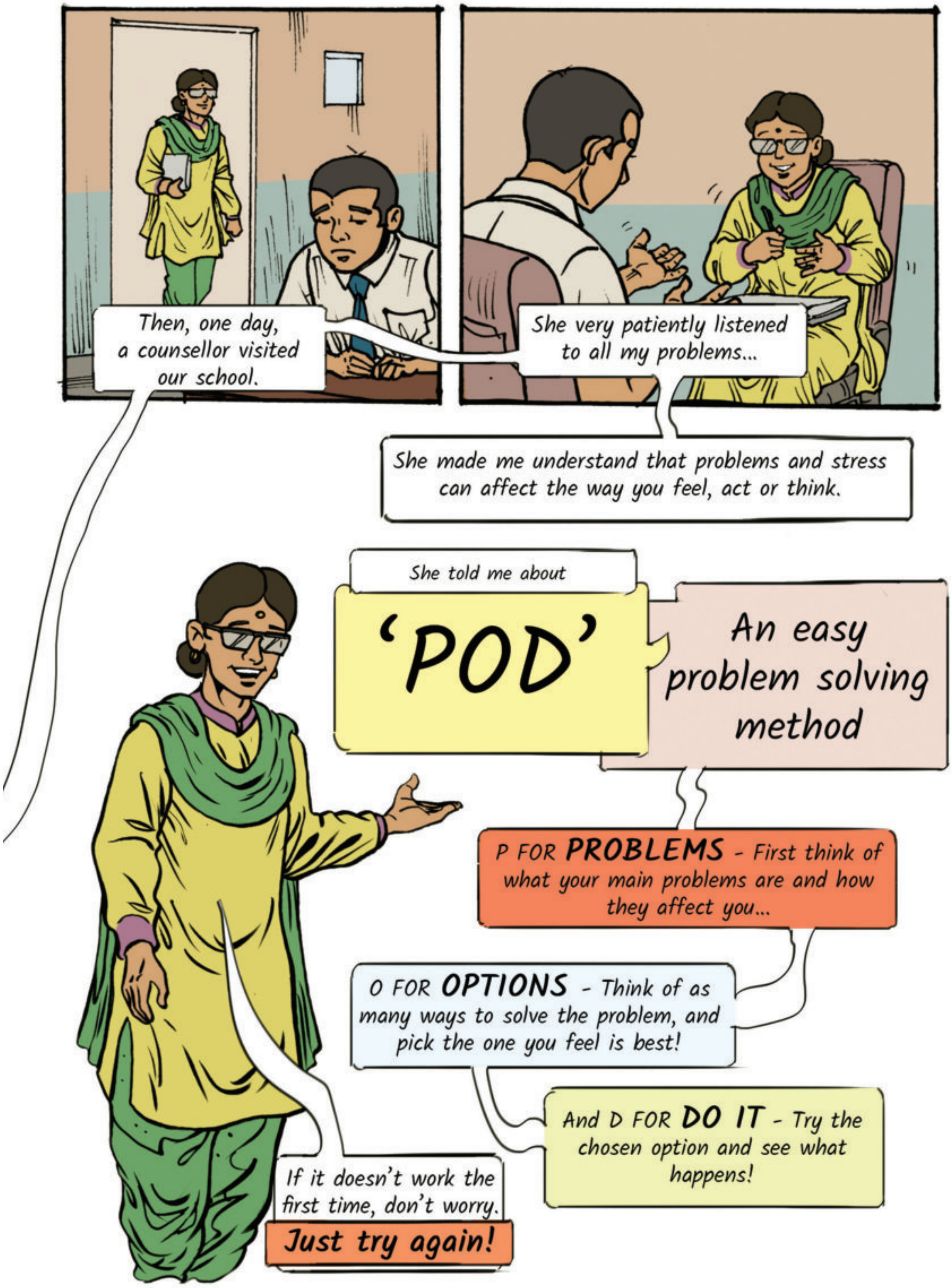
Then, one day, a counsellor visited our school.
She very patiently listened to all my problems...
She made me understand that problems and stress can affect the way you feel, act or think.
She told me about
'POD'
An easy problem solving method
P FOR PROBLEMS - First think of what your main problems are and how they affect you...
O FOR OPTIONS - Think of as many ways to solve the problem, and pick the one you feel is best!
And D FOR DO IT - Try the chosen option and see what happens!
If it doesn't work the first time, don't worry.
Just try again!

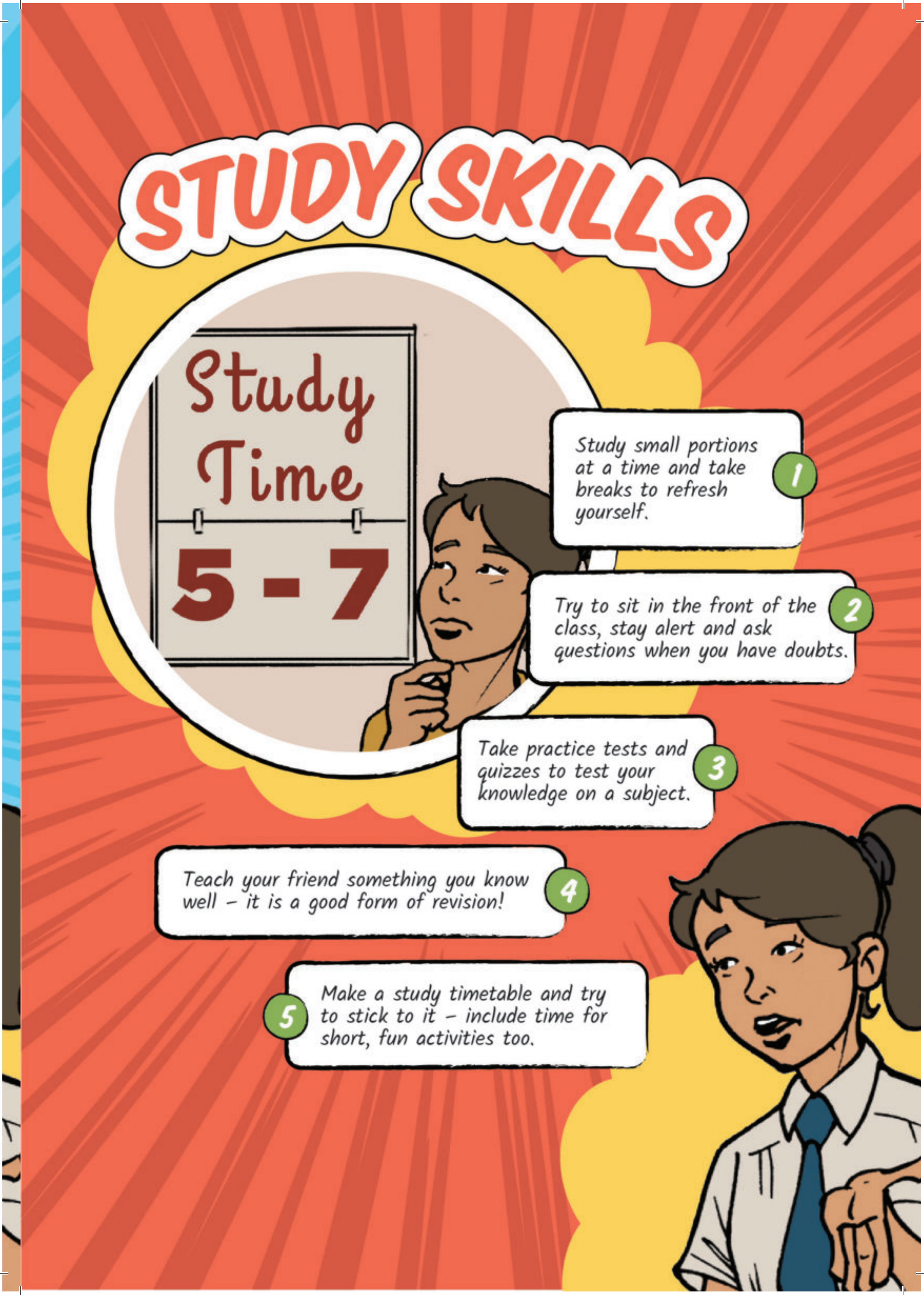
STUDY SKILLS
Study Time
5 - 7
Study small portions at a time and take breaks to refresh yourself. 1
Try to sit in the front of the class, stay alert and ask questions when you have doubts. 2
Take practice tests and quizzes to test your knowledge on a subject. 3
Teach your friend something you know well – it is a good form of revision! 4
5 Make a study timetable and try to stick to it – include time for short, fun activities too.
