# Supplementary material for: Development of a transdiagnostic, low-intensity, psychological intervention for common adolescent mental health problems in Indian secondary schools
Source: Behav Res Ther. 2020 Jul;130:103439. doi: 10.1016/j.brat.2019.103439 (PMC7322400; doi:10.1016/j.brat.2019.103439)
Supplement: Multimedia component 3 [file mmc3.pdf]

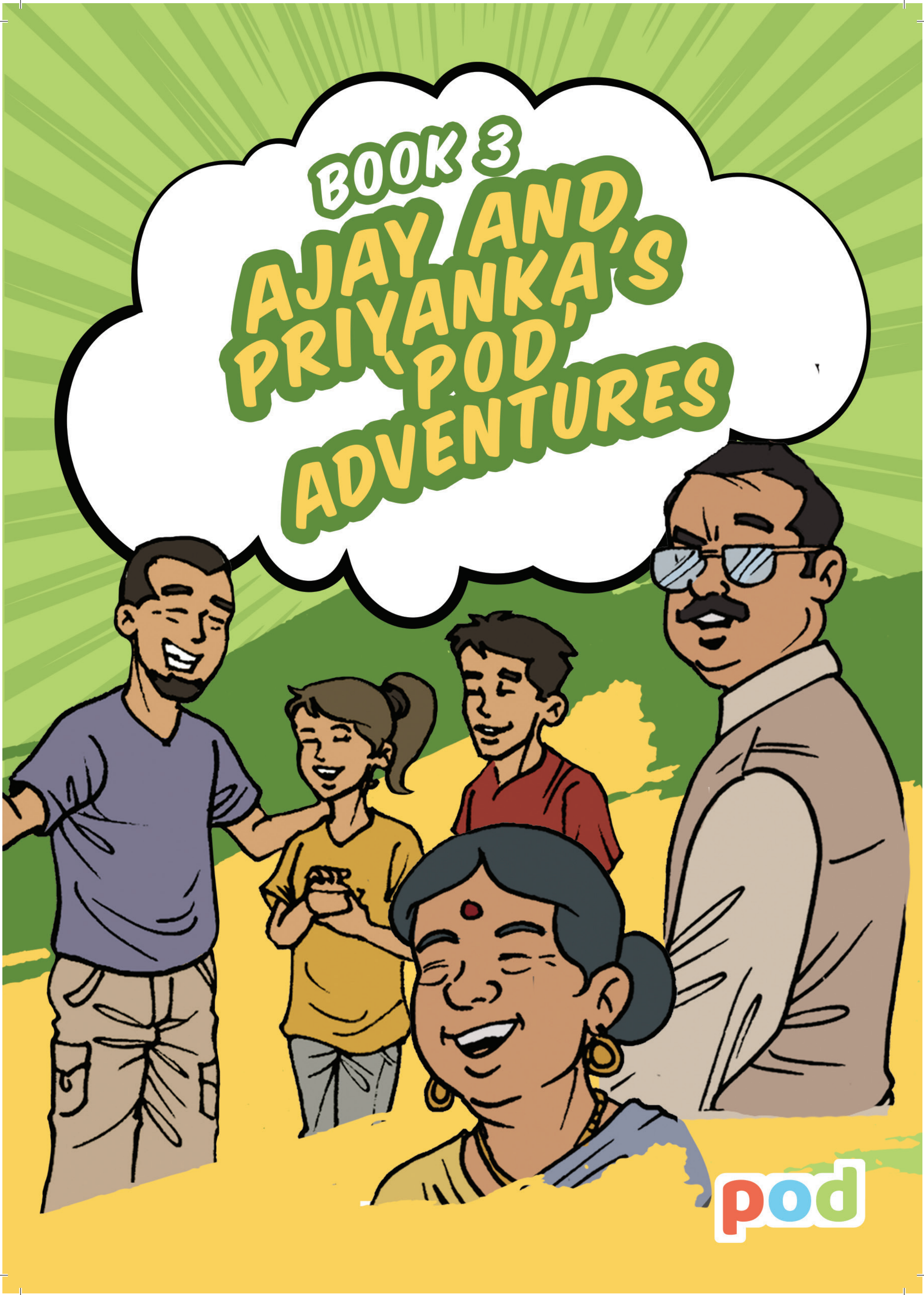
BOOK 3
AJAY AND PRIYANKA'S 'POD' ADVENTURES
pod

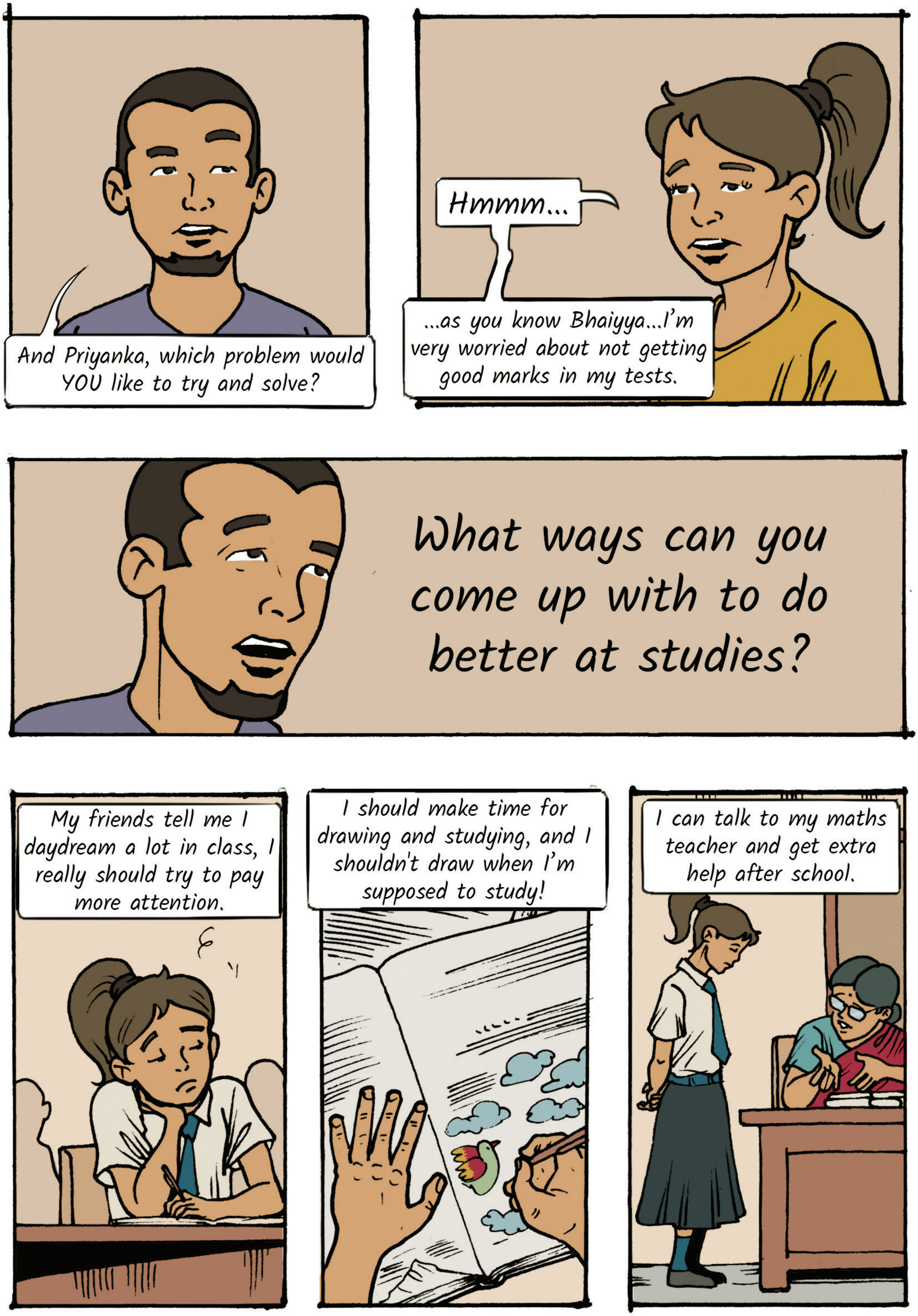
And Priyanka, which problem would YOU like to try and solve?
Hmmm...
...as you know Bhaiyya...I'm very worried about not getting good marks in my tests.
What ways can you come up with to do better at studies?
My friends tell me I daydream a lot in class, I really should try to pay more attention.
I should make time for drawing and studying, and I shouldn't draw when I'm supposed to study!
I can talk to my maths teacher and get extra help after school.

P Write down your main **Problem**.

O Write down the best **Option** you have chosen to solve your problem.

pod

Make notes on what happened when you tried your option.
How did it go? What could be improved next time?
